# Cystathionine gamma-lyase (Cth) induces efferocytosis in macrophages via ERK1/2 to modulate intestinal barrier repair

**DOI:** 10.1186/s12964-022-01030-y

**Published:** 2023-01-23

**Authors:** Xiao-Hu Zhao, Ting Yang, Meng-Yao Zheng, Peinan Zhao, Li-Ya An, Yu-Xing Qi, Ke-Qian Yi, Peng-Cheng Zhang, Da-Li Sun

**Affiliations:** 1 Department of Gastrointestinal Surgery, Second Affiliated Hospital of Kunming Medical University/Second Faculty of Clinical Medicine, Kunming Medical University, Kunming, 650101 China; 2 Department of Gastroenterology, Second Affiliated Hospital of Kunming Medical University/Second Faculty of Clinical Medicine, Kunming Medical University, Kunming, 650101 China; 3 Department of Medicine (Alfred Hospital), Central Clinical School, Monash University, 99 Commercial Rd, Melbourne, VIC 3004 Australia

**Keywords:** Efferocytosis, Cystathionine gamma-lyase (Cth), Intestinal ischaemia, Reperfusion injury (IR), Extracellular signal-regulated kinase 1/2 (ERK12), Intestinal barrier repair

## Abstract

**Background:**

The inflammatory response induced by intestinal ischaemia‒reperfusion injury (I/R) is closely associated with infectious complications and mortality in critically ill patients, and the timely and effective clearance of apoptotic cells is an important part of reducing the inflammatory response. Studies have shown that the efferocytosis by phagocytes plays an important role. Recently, studies using small intestine organoid models showed that macrophage efferocytosis could promote the repair capacity of the intestinal epithelium. However, no studies have reported efferocytosis in the repair of I/R in animal models.

**Results:**

We used an in vivo efferocytosis assay and discovered that macrophage efferocytosis played an indispensable role in repairing and maintaining intestinal barrier function after I/R. In addition, the specific molecular mechanism that induced macrophage efferocytosis was Cth-ERK1/2 dependent. We found that Cth drove macrophage efferocytosis in vivo and in vitro. Overexpression/silencing Cth promoted/inhibited the ERK1/2 pathway, respectively, which in turn affected efferocytosis and mediated intestinal barrier recovery. In addition, we found that the levels of Cth and macrophage efferocytosis were positively correlated with the recovery of intestinal function in clinical patients.

**Conclusion:**

Cth can activate the ERK1/2 signalling pathway, induce macrophage efferocytosis, and thus promote intestinal barrier repair.

**Video Abstract**

**Supplementary Information:**

The online version contains supplementary material available at 10.1186/s12964-022-01030-y.

## Introduction

In clinical practice, major surgery, severe trauma, extensive burns and severe infections can lead to a state of stress and the redistribution of blood throughout the body, leaving intestinal tissues in a state of ischaemia and hypoxia, which results in a significant increase in apoptosis in the intestinal mucosa and related lymphoid tissues and impairment of intestinal mucosal barrier function; when the blood supply is restored, this leads to more serious damage to the ischaemic area instead of restoring function. This pathophysiological process is known as intestinal ischaemia/reperfusion injury (I/R), and it is strongly associated with increased infectious complications and mortality in critically ill patients [1].

In the context of I/R, increased dead intestinal mucosal cells can exacerbate the inflammatory reaction, and the strength of the inflammatory reaction determines the severity of the I/R; therefore, the timely and effective removal of dead cells is an essential part of reducing the inflammatory reaction. clearing dead cells by phagocytes is known as efferocytosis and is important for the maintenance of growth and development, internal homeostasis and reducing inflammation, which is divided into 3 stages: recognition, engulfment and the digestion of dead cells [2, 3]. Several studies have shown that efferocytosis can induce positive effects on inflammation, clotting clearance and tissue repair in the heart, lungs, liver, kidneys and brain [4–7]. However, studies on the molecular mechanism of efferocytosis in the resolution of I/R and intestinal barrier repair are lacking. There have only been a few in vitro studies on efferocytosis in intestinal cells, one showing that efferocytosis could result in the synthesis of the anti-inflammatory mediator PGE_2_ and then enhance Th17 immunity and host defence during infectious colitis [8] and another focusing on whether Panther cells have a phagocytic-like efferocytosis effect [9]; however, neither study examined the role of efferocytosis in intestinal mucosal barrier repair. Only one recent study using a small intestine organoid model, which focused on the repair of the intestinal mucosal barrier by macrophage efferocytosis, showed that cyclooxygenase 2 (COX2) in macrophages could mediate efferocytosis and resolution reprogramming and promote intestinal epithelial repair [10]. However, no studies have examined efferocytosis in the repair of intestinal I/R in vivo, and an important reason for this is related to the lack of validated indicators of the level of efferocytosis in the intestinal mucosa.

Cystathionine gamma-lyase (Cth or CSE) is an enzyme that can break down cystathionine into cysteine, alpha-ketobutyric and ammonia, which are intermediate metabolites that eventually catalyse the synthesis of gases such as H_2_S. It has been reported that the amount of H_2_S is associated with diseases such as hypertension, diabetes, acute pancreatitis and haemorrhagic shock [11, 12]. Currently, Cth regulation of the intestinal mucosal barrier has been relatively poorly studied. In the context of intestinal barrier damage due to inflammation, some researchers have found that Cth can affect the function of the intestine and intestinal microecology by influencing the synthesis of H_2_S and therefore treat colitis [13, 14]. In a study of FXR-mediated protection of intestinal mucosal barrier function, it was found that the protective effect of FXR was due to the mediation of Cth [15].

Recent basic studies have discovered that the extracellular signal-regulated kinase 1/2 (ERK1/2) signalling pathway may be involved in the regulation of efferocytosis. One study showed that apoptotic cell-derived nucleotides induced efferocytosis and thus promoted macrophage proliferation without involving Mertk-ERK1/2 and mTORC2 [16]. Apoptotic cell-derived methionine promotes tissue resolution during efferocytosis via the ERK1/2 phosphatase Dusp4 [17], and the angiotensin-(1–7)/MasR axis promotes the migration of monocytes/macrophages to perform efferocytosis in a MEK/ERK1/2-dependent manner [18]. In addition, a study showed that H_2_S could elongate primary cilia on kidney tubular epithelial cells by activating ERK, which suggests that Cth can regulate the function of the ERK1/2 pathway via H_2_S [19].

Therefore, in this study, we first designed and validated a method for the in vivo detection of the intestinal mucosal efferocytosis index to verify the involvement of efferocytosis in intestinal mucosal barrier injury repair during I/R, examined the genes involved in this process by transcriptome sequencing, and then verified that Cth regulated efferocytosis through the ERK1/2 signalling pathway to provide a theoretical basis for exploring the molecular mechanism and possible targets of I/R repair.

## Results

### Efferocytosis under I/R conditions occurs and produces an anti-inflammatory repair phenotype in vitro and in vivo

**Fig. 1:**
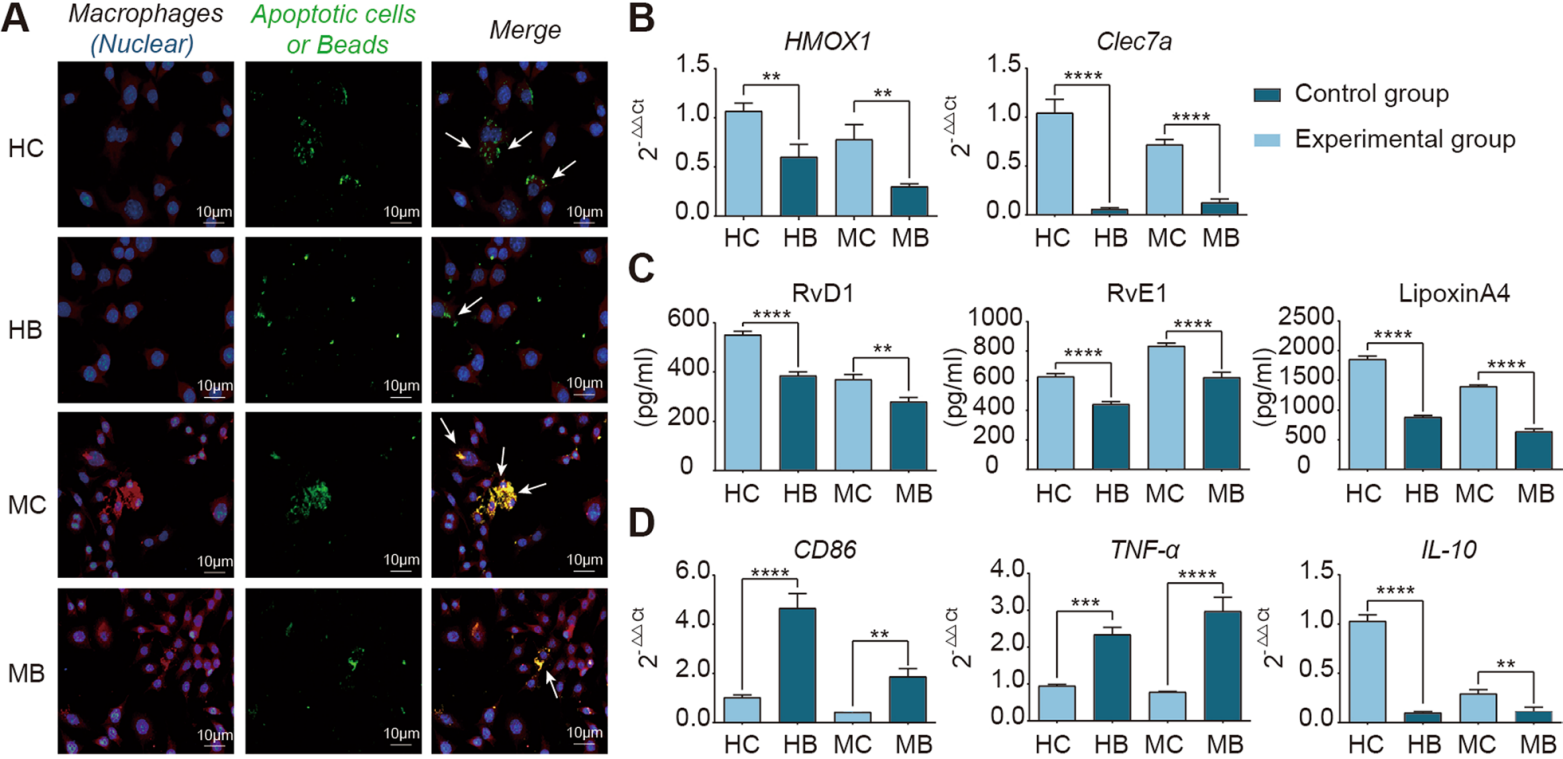
In vitro, the engulfment of apoptotic endothelial cells can induce a reparative phenotype in macrophages. **A** Representative immunofluorescence images showing macrophage engulfment of apoptotic endothelial cells or hydroxylate beads in vitro. CellTracker™ Deep Red Dye labels macrophages (red); CellTracker™ Green BODIPY™ Dye labels endothelial cells and hydroxylate beads (green). **B** Changes in reparative phenotypic indicators (Hmox1 and Clec7a) after macrophage engulfment of apoptotic endothelial cells or hydroxylate beads. **C** Changes in SPMs (RvD1, RvE1, Lipoxin A4) after macrophage engulfment of apoptotic endothelial cells or hydroxylate beads. **D** Changes in the gene expression of inflammatory factors (CD86, TNF-α, IL-10). HC: Coculture of human-derived macrophages with apoptotic Caco-2 cells; HB: Coculture of human-derived macrophages with apoptotic Caco-2 cells; MC: Coculture of murine-derived macrophages with apoptotic IEC-6 cells; MB: Coculture of murine-derived macrophages with hydroxylate beads. Experimental group versus control group; Student’s test. **P ≤ 0.01, ***P ≤ 0.001, ****P ≤ 0.0001

Patients who undergo major surgery, trauma and severe infection inevitably suffer from intestinal ischaemic and hypoxic injury, and clinical evidence suggests that intestinal I/R is strongly associated with increased infectious complications and mortality in patients [1]. Therefore, the timely repair of intestinal barrier damage after I/R is particularly important. Previous studies have reported that efferocytosis plays an important role in maintaining the growth and development of an organism, internal homeostasis and inflammation regression [2]. To clarify the necessity of phagocytosis to engulf apoptotic cells for the anti-inflammatory repair phenotype, we designed in vitro experiments in which macrophages and apoptotic endothelial cells were cocultured. In the experimental group, we used primary cultured mouse bone marrow-derived macrophages and THP-1-cell-induced human-derived macrophages cocultured with apoptotic IEC-6 and Caco-2 cell lines under ischaemic and hypoxic environments, respectively (Fig. 1, Additional file 1: Figure S1). To determine whether nonspecific macrophage phagocytosis would have the same effect, we incubated macrophages with hydroxylate beads as a control. Phagocytes had a stronger phagocytic capacity for apoptotic cells than hydroxylate beads in murine-derived macrophages cocultured with apoptotic IEC-6 cells and in the human-derived macrophages cocultured with apoptotic Caco-2 cells (Fig. 1A). Furthermore, we found that in the group of murine-derived macrophages cocultured with apoptotic IEC-6 cells and in the group of human-derived macrophages cocultured with apoptotic Caco-2 cells, the repair markers haem oxygenase-1 (Hmox1) and dectin-1 (Clec7a) [4, 20], the specialized pro-resolving mediators (SPMs) RvD1, RvE1, and LipoxinA4 [5, 21] and the anti-inflammatory factor IL-10 [22] were higher than those in the macrophage and hydroxylate bead coculture group (Fig. 1B–D), while the proinflammatory factors CD86 and TNF-α were lower than those in the macrophage and hydroxylate bead coculture group (Fig. 1D). These results suggest that efferocytosis is necessary for producing anti-inflammatory and repair phenotypes.

**Fig. 2:**
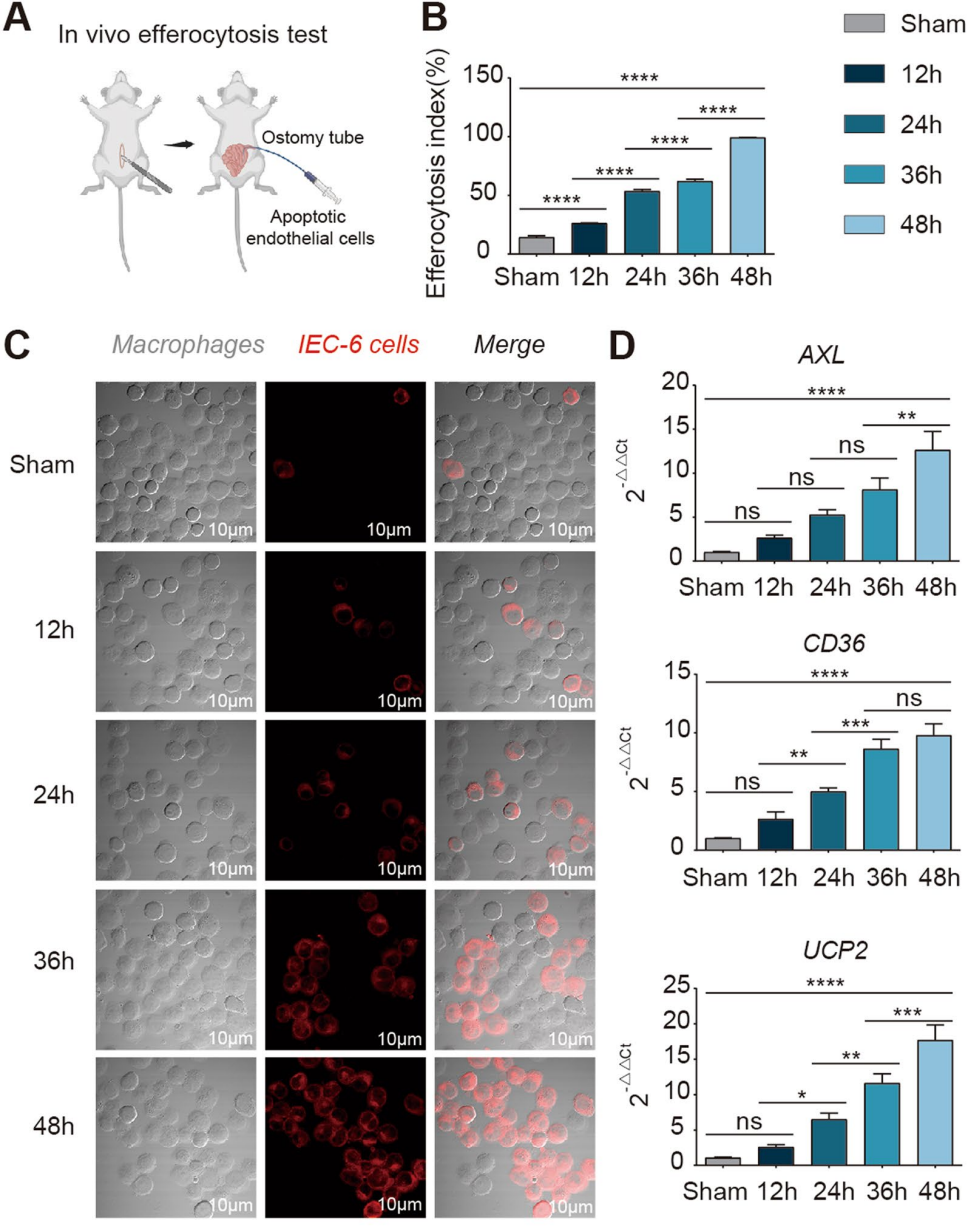
In vivo, efferocytosis was present in the damaged intestine, and its level gradually increases with prolonged I/R time. **A** Schematic diagram of the in vivo efferocytosis assay. **B** Changes in the efferocytosis index (proportion of macrophages engulfing apoptotic endothelial cells to total macrophages). **C** Representative immunofluorescence images showing macrophage phagocytosis of apoptotic endothelial cells in the intestine of live animal models. CellTracker™ Deep Red Dye labels apoptotic IEC-6 cells (red); F4/80 PE antibody labels macrophages. **D** Gene expression of efferocytosis biomarkers (AXL, CD36, UCP2). Sham: sham-operated group; 12 h: 12-h post-I/R group; 24 h: 24-h post-I/R group; 36 h: 36-h post-I/R group; 48 h: 48-h post-I/R group. n = 6/group. *P ≤ 0.05, **P ≤ 0.01, ***P ≤ 0.001, ****P ≤ 0.0001, one-way ANOVA with Tukey’s multiple comparisons test

For in vivo animal models, it is still unclear whether efferocytosis occurs inside the intestine after I/R; therefore, we used an in vivo intestinal efferocytosis detection technique (Fig. 2A, Methods). We found that the phagocytosis of dead cells by macrophages was gradually enhanced after I/R (Fig. 2C), the efferocytosis index was gradually increased (Fig. 2B, Additional file 2: Figure S2), and the efferocytosis markers AXL, CD36 and UCP2[4, 5, 23, 24] were gradually increased with prolonged recovery time after I/R (Fig. 2D). These results suggest that the phenotype of efferocytosis is not only present within the intestine but also gradually increases with prolonged injury repair time. Next, we further investigated the mechanism by which efferocytosis promotes intestinal barrier repair in I/R.

**Fig. 3:**
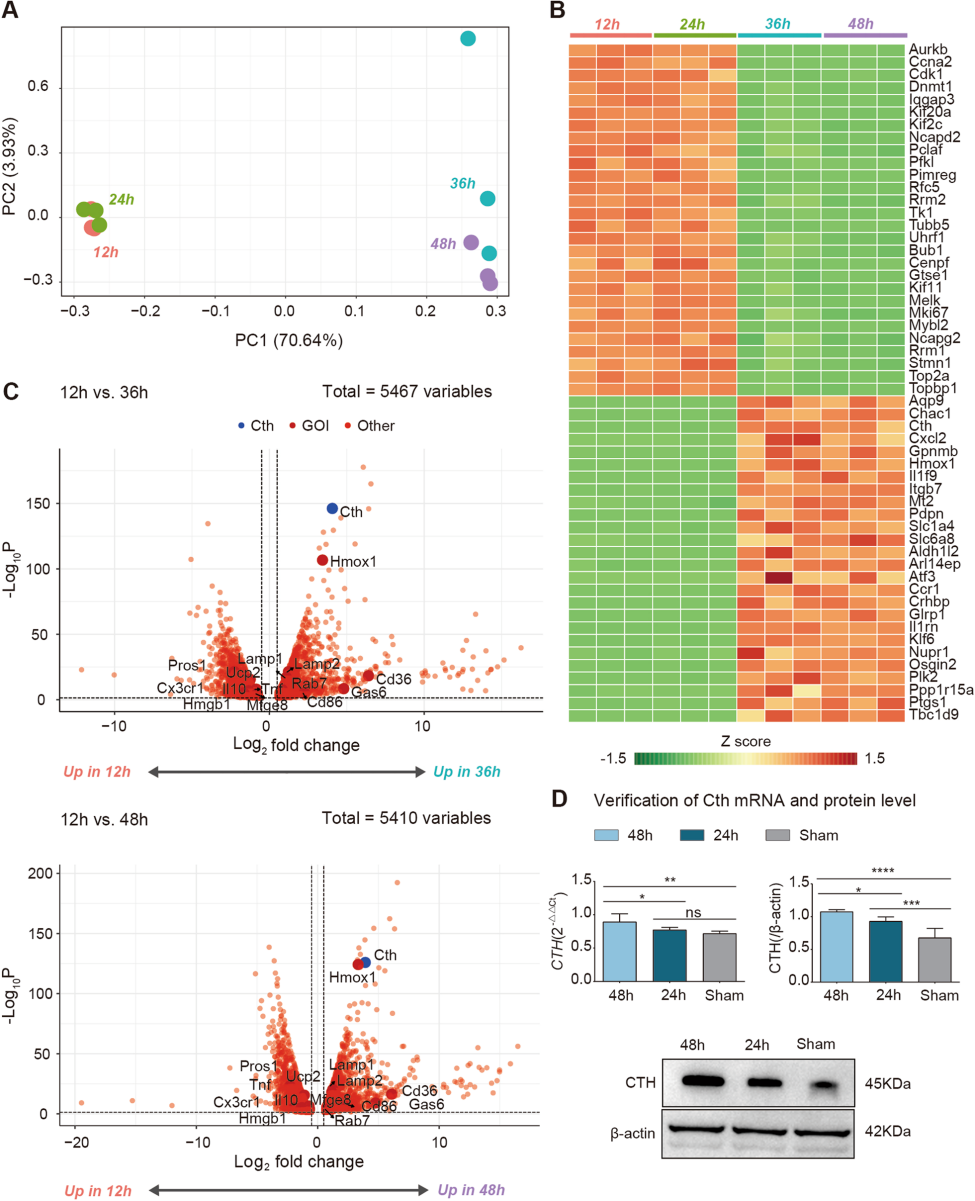
Transcriptome sequencing analysis identified Cth as one of the potential mediators of efferocytosis and the resolution phase after I/R. **A** PCA plot: PCA showing the clustering of the RNA-seq data of mouse intestinal macrophages after intestinal ischaemia‒reperfusion surgery. Macrophages at 36 and 48 h postsurgery were differentiated from those in the first 24 h. PCA was performed on a normalized gene count matrix (FPKM) using DESeq2. **B** Heatmap: Heatmap showing the 54 most significantly differentiated genes (− log10(*P* value) > 70) at 12 h and 48 h postsurgery. Colours indicate the gene expression z score. Z normalization was performed on the FPKM for each gene. **C** Volcano plot, top: Volcano plot showing 5467 genes expressed in postsurgery intestinal macrophages. Dashed lines represent the thresholds (*P* value < 0.05, log2-fold change > 0.5) for significantly DEGs at 12 h and 36 h after surgery. Cth and a list of known efferocytosis marker genes are labelled. Bottom: Volcano plot showing 5410 genes expressed in postsurgery intestinal macrophages at 12 h and 36 h after surgery. Cth and a list of known efferocytosis marker genes are labelled. **D** Bar graphs and western blots showing the mRNA and protein expression levels of Cth in the sham-operated group (Sham), the 24-h post-I/R group (24 h) and the 48-h post-I/R group (48 h). n = 6/group. *P ≤ 0.05, **P ≤ 0.01, ***P ≤ 0.001, ****P ≤ 0.0001, one-way ANOVA with Tukey’s multiple comparisons test

*The expression of a large range of genes is altered after I/R-induced efferocytosis*. To better understand the genetic alterations in macrophages after I/R, we performed transcriptome sequencing analysis in an animal model after macrophages were selected by flow cytometry (Fig. 3). Principal component analysis (PCA) showed well-stratified samples between the 12-h group and the 24-h, 36-h and 48-h groups after I/R surgery (Fig. 3A). We then used a heatmap to visualize the change in the expression of 54 genes that were most significantly different at 12 h and 24 h after I/R (Fig. 3B), including 28 genes that were downregulated with prolonged I/R time, which were mostly associated with the cell cycle (such as Ccna2, Cdk1, Dnmt1, Uhrf1, Bub1, Cenpf, Gtse1Mybl2, Aurkb*)*, the cell proliferation marker Mki67 and the kinesin family molecules Kif20a, Kif2c and Kif11. This is probably because the intestine is in a state of ischaemia and hypoxia that affects cell division and differentiation. There were also 26 genes that were upregulated with prolonged I/R time, including Aqp9, Chac1, Cth, Hmox1, Cxcl2, Gpnmb, IL1f9, Itgb7, Atf3, IL1rn, Ccr1, Nupr1, Plk2, and Ptgs1. Many of these genes can play protective roles, such as the chemokine family member Cxcl2, which is also known as macrophage inflammatory protein 2-alpha (MIP2-α) and growth-regulated protein beta (Gro-β), which is a powerful neutrophil granulocyte chemotactic agent involved in many immune responses, including wound healing [25]. In addition, IL1rn, which is also called interleukin-1 receptor antagonist (IL-1RA), is a natural inhibitor of the proinflammatory factor IL-beta that is elevated with increasing I/R time [26]. Other upregulated genes can also play protective roles by participating in the anti-inflammatory response, such as Aqp9 [27], Hmox1 [20] and Plk2 [28]. Therefore, we focused on these genes that are upregulated with longer I/R times.

Next, to identify differentially expressed genes (DEGs) that could drive macrophage efferocytosis, we compared gene expression at 12 h post-I/R (when efferocytosis is just occurring according to our previous study) and 36 h post-I/R (when efferocytosis is transitioning to a beneficial phenotype in this model), as well as 12 h post-I/R and 48 h post-I/R (when efferocytosis effects are maximal), and as with the heatmap analysis, we identified many DEGs after the two sets of comparisons (Fig. 3C). Interestingly, we found that in I/R, marker molecules of efferocytosis (UCP2 and Cd36) [5, 23, 24], molecules that contribute to macrophage maturation and phagosome formation (Lamp1, Lamp2 and Rab7), ‘find me signals’ (Cx3cr1, Hmgb1), ‘eat me signals’ (Pros1, also called Proteins), ‘bridging molecules’ (Mfge8 and Gas6) during efferocytosis [2], the anti-inflammatory factor IL-10 and the repair indicator Hmox1 were significantly different. The transcriptome analysis results suggest that efferocytosis plays an important role in regulating the macrophage phenotype after I/R and that there is a potential link between intestinal barrier protection and mitigation of the macrophage inflammatory response.

More importantly, we also found that the expression of Cth, a key enhancer of macrophage activation [29], was significantly upregulated after I/R surgery. Combined with the PCA results and heatmap analyses, these results suggest that Cth may be a key gene that drives macrophage efferocytosis after I/R. To verify these results, we examined Cth expression in the 24-h post-I/R group, the 48-h post-I/R group and the sham-operated group using PCR and western blotting and found that there was a significant difference in the gene and protein expression in the post-I/R group compared to the sham-operated group, and there was an increase with increasing I/R time (Fig. 3D). This finding is consistent with the results of our transcriptome analysis. Therefore, we hypothesize that Cth can alter the macrophage phenotype, supports efferocytosis, and has an important role in the repair of intestinal barrier injury.

### Cth regulates intestinal inflammation and intestinal barrier injury recovery via the ERK1/2 pathway

**Fig. 4:**
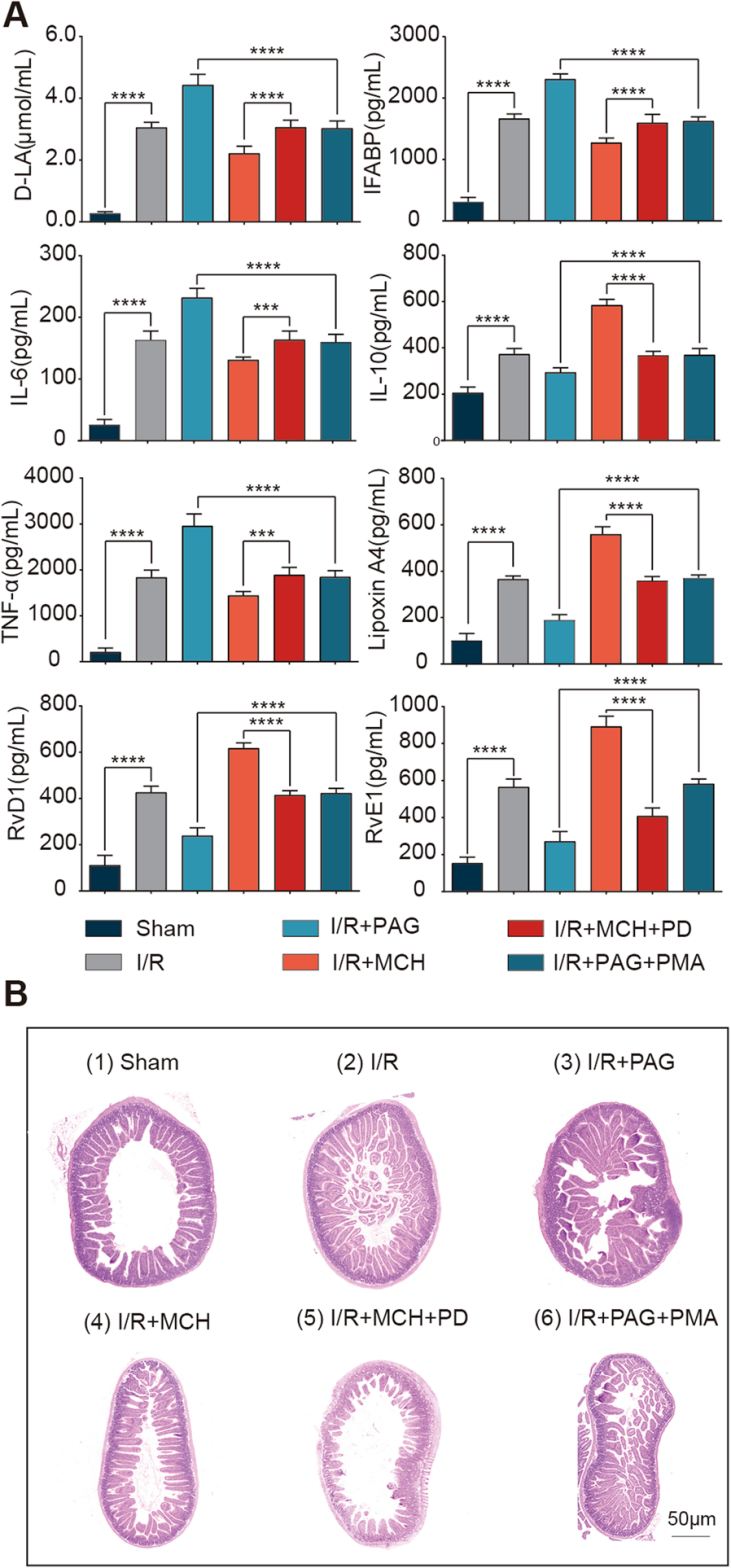
Changes in Cth affect intestinal barrier repair, and the mechanism is dependent on the ERK1/2 pathway in vivo. **A** Bar graphs showing changes in intestinal barrier indicators (D-LA, IFABP), inflammatory factors (IL-6, TNF-α) and anti-inflammatory repair indicators (IL-10, Lipoxin A4, RvD1, RvE1) following alterations in the Cth and ERK1/2 pathways. n = 6/group. ***P ≤ 0.001, ****P ≤ 0.0001, one-way ANOVA with Tukey’s multiple comparisons test. **B** Representative H&E staining images showing changes in intestinal mucosal morphology after alterations in the Cth and ERK1/2 pathways (scale = 50 μm). Sham: sham-operated group; I/R: I/R group; I/R + PAG: I/R model mice were administered the Cth inhibitor propargylglycine (PAG); I/R + MCH: I/R were administered the Cth agonist methacholine (MCH); I/R + MCH + PD: I/R model mice were administered the Cth agonist MCH and the ERK1/2 inhibitor PD98059 (PD); I/R + PAG + PMA: I/R model mice were administered the Cth inhibitor PAG and the ERK1/2 agonist PMA

As Cth may be the key molecule that induces macrophage efferocytosis, which in turn results in anti-inflammatory and intestinal repair phenotypes, we next explored the potential mechanisms. ERK1/2, which is a member of the mitogen-activated protein kinase (MAPK) family, has been reported to be associated with cell survival, proliferation and development [30] and plays an important role in gastric mucosal cell proliferation and enhancing intestinal epithelial cell barrier function [31, 32]. In addition, ERK1/2 is one of the important pathways for enhancing macrophage phagocytosis, promoting efferocytosis and suppressing inflammation [33, 34]; in addition, Cth overexpression can induce a sustained increase in ERK activity [12]. Thus, we decided to investigate whether Cth regulates efferocytosis through the ERK1/2 signalling pathway to promote intestinal barrier damage repair. First, we explored whether Cth affects the anti-inflammatory and intestinal barrier damage repair phenotypes using an animal model. Intestinal fatty acid binding protein (I-FABP) is a low molecular mass cytoplasmic protein that is expressed only in intestinal epithelial cells throughout the small intestine and part of the colon, and when the intestine is damaged, I-FABP is rapidly released from mature intestinal epithelial cells into the blood; thus it is considered a marker of intestinal epithelial cell injury [35]. In addition, D-lactate is metabolized by the intrinsic intestinal flora and cannot be produced and metabolized by other tissues in the body, and so it can also respond to changes in intestinal permeability and can also be used as an indicator of intestinal barrier damage [36]. Thus, we could see that when mice undergo I/R injury, the functional indicators of intestinal barrier damage (I-FABP and D-lactate) were significantly higher compared to those in the sham-operated group, and when I/R mice were given intraperitoneal injections of the Cth agonist MCH[37], there was a decreasing trend; in contrast, I-FABP and D-lactate were elevated when the mice were given intraperitoneal injections of the Cth inhibitor PAG [38] (Fig. 4A). Similarly, the Cth agonist MCH suppressed the increases in the proinflammatory markers IL-6 and TNF-α after I/R injury compared to those in the sham-operated group, while the Cth inhibitor PAG slowed this trend. In addition, the changes in IL-10 and RvD1, RvE1 and LipoxinA4, which play protective and reparative roles, were another way to verify that Cth affected the anti-inflammatory factors and repair phenotype of the intestine (Fig. 4A). Moreover, we observed morphological changes in the intestinal mucosa in the mice and found that the arrangement of intestinal mucosal villi was disorganized after I/R, with clumped villi in the middle, and injury was more severe than that in the sham-operated group. The villi were more disorganized when the Cth inhibitor PAG was administered, while the arrangement of villi became slightly regular when the Cth agonist MCH was administered (Fig. 4B). These results suggest that changes in Cth affect the anti-inflammatory and intestinal barrier damage repair phenotype of the intestine.

Next, we further validated whether the protective effect of Cth was dependent on the ERK1/2 pathway in vitro and in vivo. After the I/R model group was administered intraperitoneal injections of the Cth inhibitor PAG followed by the ERK agonist PMA[39], the increases in the intestinal barrier damage indicators D-lactate and I-FABP and proinflammatory factors IL-6 and TNF-α were reversed, while the decreases in the anti-inflammatory factors IL-10 and SPMs (RvD1, RvE1, and LipoxinA4) were significantly increased. In contrast, after intraperitoneal administration of the Cth agonist MCH and the ERK inhibitor PD98059 [40, 41] in I/R model mice, the decreases in the intestinal barrier damage indicators D-lactate and I-FABP and the proinflammatory markers IL-6 and TNF-α were significantly increased, while the increases in the anti-inflammatory factors IL-10 and SPMs (RvD1, RvE1 and Lipoxin A4) were significantly decreased (Fig. 4A). Similarly, morphological changes in the intestinal mucosa showed a similar trend (Fig. 4B). In vitro, we overexpressed and silenced Cth in a mouse peritoneal macrophage cell line (RAW 264.7) in a coculture system (Additional file 3: Figure S3) and then added the ERK inhibitor PD98059 and the ERK agonist PMA. These treatment did not affect the cells (Fig. 7A, Additional file 5: Figure S5), but when macrophages overexpressing Cth in the coculture system were treated with the ERK inhibitor PD98059, the proinflammatory factors IL-10 and SPMs (RvD1, RvE1 and Lipoxin A4) were suppressed, while the expression of IL-10 and RvD1, RvE1, LipoxinA4 were elevated in Cth-silenced cells after the addition of the ERK agonist PMA (Fig. 7B, C). These results demonstrated that Cth regulates intestinal inflammation and that intestinal barrier damage repair is dependent on the ERK1/2 pathway.

### The Cth-dependent ERK1/2 pathway regulates macrophages, resulting in enhanced efferocytosis

**Fig. 5:**
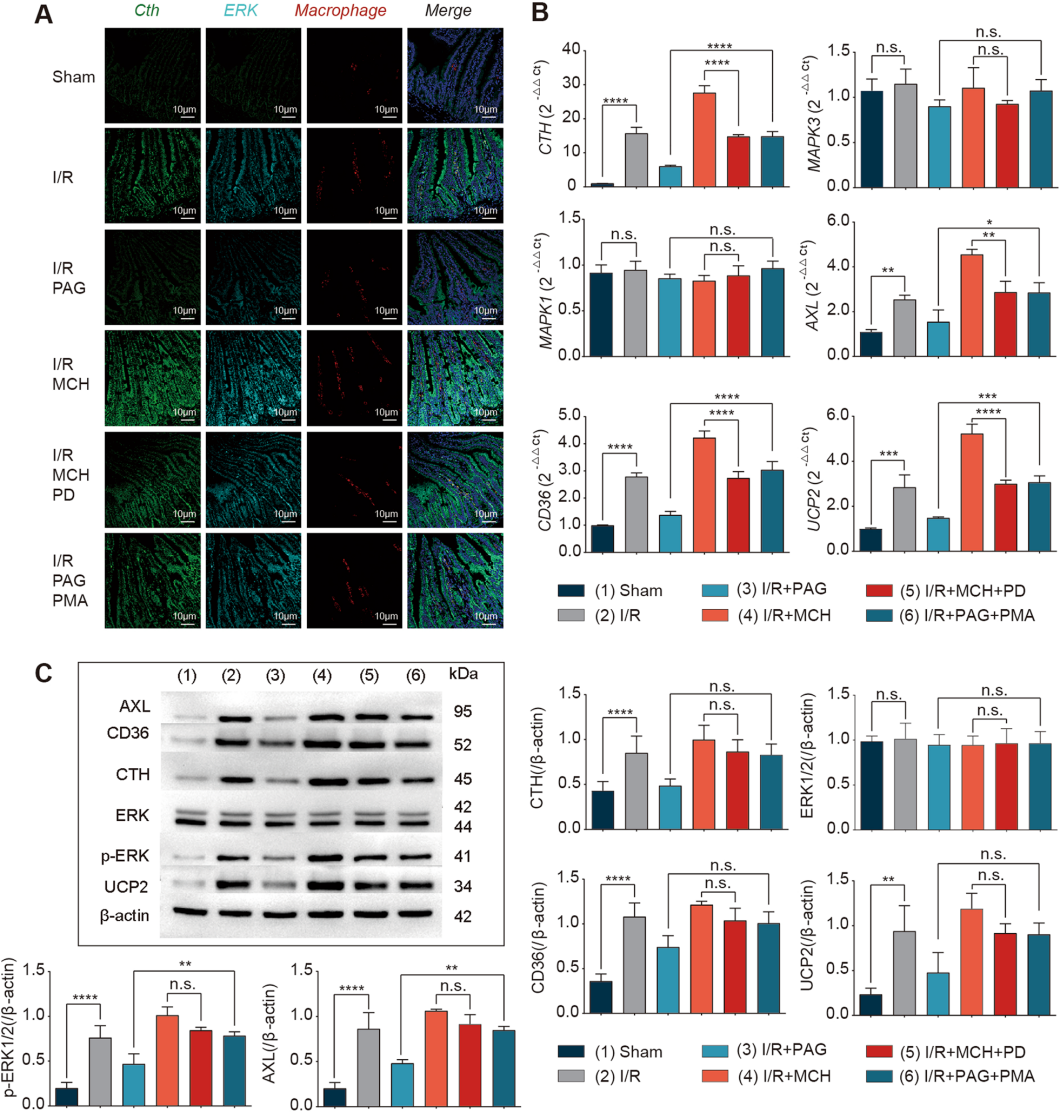
Cth induces macrophage efferocytosis in an ERK1/2-dependent manner after I/R. **A** Representative immunofluorescence images showing the colocalization of Cth, ERK1/2 and macrophages following alterations in Cth and ERK1/2. Rabbit anti-CTH/FITC labels Cth (green); rabbit anti-phospho-ERK1/2/PE labels ERK1/2 (cyan); APC anti-F4/80 labels macrophages (red). **B, C** The levels of Cth, ERK1/2 and the biomarkers of efferocytosis (AXL, CD36, UCP2) were measured using q-PCR and western blotting. n = 6/group. *P ≤ 0.05, **P ≤ 0.01, ***P ≤ 0.001, ****P ≤ 0.0001, one-way ANOVA with Tukey’s multiple comparisons test. The subgroups and interventions were identical to those in Fig. 4

To clarify the potential mechanism by which Cth regulates efferocytosis, we first examined the effect of Cth on the ERK1/2 pathway and efferocytosis in a live mouse model. When the Cth inhibitor PAG was administered to the I/R model, the PCR results showed that Cth was significantly inhibited at the gene level compared to that in the control group, but there was no effect on ERK1/2 (Mapk1/3); the biomarkers of efferocytosis (Axl, CD36, UCP2) were also significantly inhibited (Fig. 5B). At the protein level, when the Cth inhibitor was administered, Cth and the biomarkers of efferocytosis (Axl, CD36, UCP2) were significantly inhibited, but only ERK1/2 phosphorylation was inhibited (Fig. 5C, D). After intraperitoneal injection of the Cth inhibitor PAG and the readministration of the ERK agonist PMA, the decreases in Cth and the biomarkers of efferocytosis (Axl, CD36, UCP2) was reversed at the gene and protein levels, and the only change in ERK1/2 occurred at the phosphorylation level (Fig. 5B–D). To further explore the relationship between Cth, ERK1/2 and macrophages, we performed fluorescence in situ hybridization (FISH) triple-labelling colocalization analysis, in which Cth was stained green, ERK1/2 was stained cyan, and macrophages were stained red. It was evident that after the administration of the Cth inhibitor PAG, green (Cth) was significantly inhibited, and cyan (ERK1/2) and red (functioning macrophages) were also inhibited, while these factors were significantly enhanced when the ERK1/2 agonist PMA was administered (Fig. 5A).

In addition, when the Cth agonist MCH was administered to I/R model mice, the gene and protein expression of Cth and the biomarkers of efferocytosis (Axl, CD36, UCP2) were significantly increased compared to those in the control group. Similarly, only p-ERK1/2 showed a significant increase. When the ERK1/2 inhibitor PD98059 was administered to I/R model mice that had already been injected intraperitoneally with the Cth agonist MCH, the original increasing trend in Cth, p-ERK, Axl, CD36, and UCP2 was reversed to a decreasing trend (Fig. 5B–D). This was similarly demonstrated by the detection of the in vivo efferocytosis index (Additional file 7: Figure S7). Likewise, the FISH results were consistent (Fig. 5A). In the live mouse model, these results suggest that the Cth-regulated efferocytosis phenotype in macrophages is dependent on the ERK1/2 pathway.

**Fig. 6:**
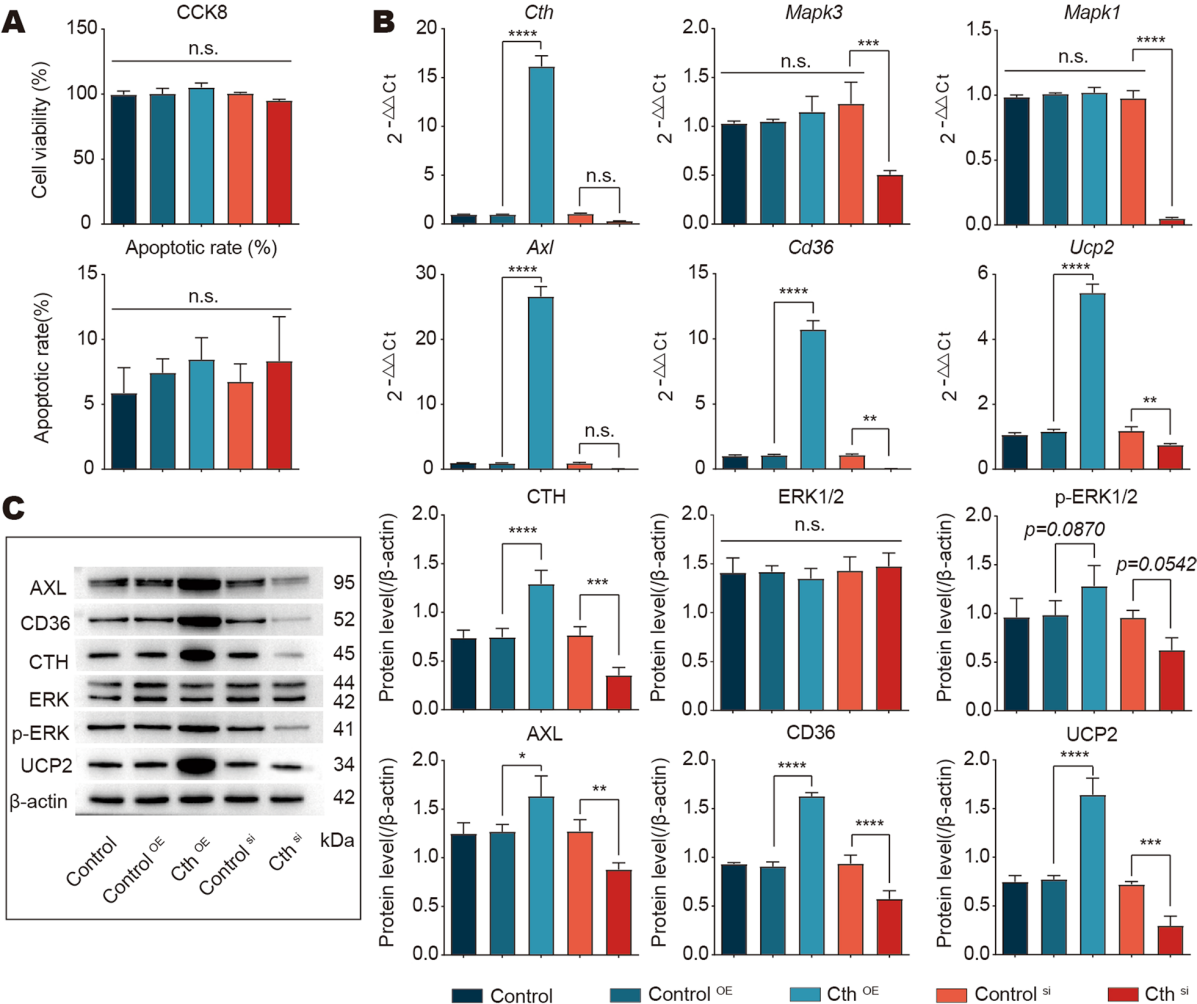
Cth silencing or overexpression in macrophages leads to downstream changes in ERK1/2 and an efferocytosis phenotype in vitro. **A** Bar graphs showing no effect on cell status after cth silencing or overexpression in macrophages. Top: The CCK8 results showing the changes in viability in each group of cells. CCK8, cell counting kit-8. Bottom: Flow cytometry showing the mortality of each group of cells. **B, C** Bar graphs showing changes in the gene and protein levels of the ERK1/2 pathway and efferocytosis biomarkers (AXL, CD36, UCP2) after alterations in Cth. Control: Mouse peritoneal macrophages; Control^OE^: Control overexpression of mouse peritoneal macrophages; Cth^OE^: Cth overexpression in mouse peritoneal macrophages; Control^si^: Cth silencing in mouse peritoneal macrophages; Cth^si^: Control silencing in mouse peritoneal macrophages. *P ≤ 0.05, **P ≤ 0.01, ***P ≤ 0.001, ****P ≤ 0.0001, one-way ANOVA with Tukey’s multiple comparisons test

In an in vitro coculture system of peritoneal macrophages (RAW 264.7) with apoptotic endothelial cells, various treatments of the cells had no effect on cell viability or apoptosis (Fig. 6A, Additional file 4: Figure S4); however the Cth overexpression group of RAW 264.7 showed significantly increased expression of Axl, CD36 and UCP2 compared to the control group at the gene and protein levels, and ERK1/2 pathway was activated, showing a significant increase in phosphorylation levels (Fig. 6B, C). These results suggest that Cth-mediated regulation of efferocytosis is dependent on the phosphorylation of ERK1/2 in vitro.

### Cth depends on macrophage efferocytosis to regulate the repair phenotype

**Fig. 7:**
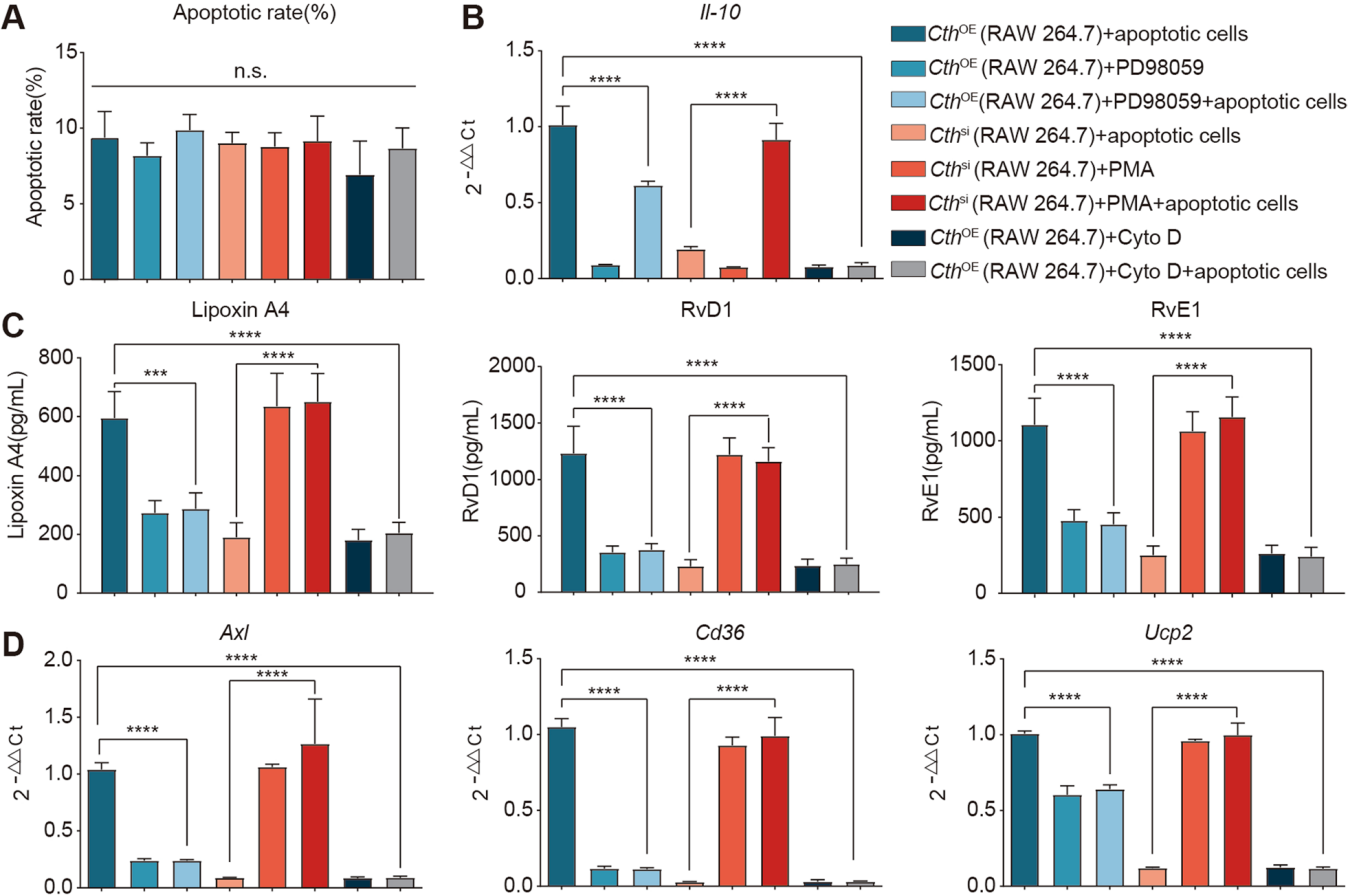
Cth regulates efferocytosis and reparative phenotypes via activation of the ERK1/2 pathway in vitro. **A** Flow cytometry showing the mortality of each group of cells after the interventions. **B** Changes in the expression of the anti-inflammatory factor IL-10. **C** Changes in the reparative phenotypic indicators SPMs (Lipoxin A4, RvD1, RvE1), as detected by ELISA. **D** Changes in efferocytosis biomarkers (AXL, CD36, UCP2), as detected by q-PCR. RAW 264.7: Mouse peritoneal macrophage cell line; Cth^OE^: Cth overexpression in RAW 264.7 cells; Cth^si^: Control silencing in RAW 264.7 cells; PD98059: ERK1/2 inhibitor; PMA: ERK1/2 agonist; Cyto D: efferocytosis inhibitor (Cytochalasin D). *P ≤ 0.05, **P ≤ 0.01, ***P ≤ 0.001, ****P ≤ 0.0001, one-way ANOVA with Tukey’s multiple comparisons test

Cytochalasin D is an inhibitor of actin polymerization and phagocytosis, and some studies have reported its use as an inhibitor of efferocytosis [42]. Therefore, in the in vitro coculture experiment, we added cytochalasin D to Cth-overexpressing peritoneal macrophages and apoptotic endothelial cells, and after excluding its effect on cell viability (Fig. 7A, Additional file 5: Figure S5), we found that efferocytosis markers (AXL, CD36, UCP2), which had been originally highly expressed, were significantly decreased (Fig. 7D). The high levels of IL-10 and the related repair molecules RvD1, RvE1, and Lipoxin A4 were also significantly decreased (Fig. 7B, C). These results suggest that Cth-mediated regulation of the repair phenotype is efferocytosis dependent.

### Cth, ERK1/2 pathway activation and efferocytosis were clinically validated

**Fig. 8:**
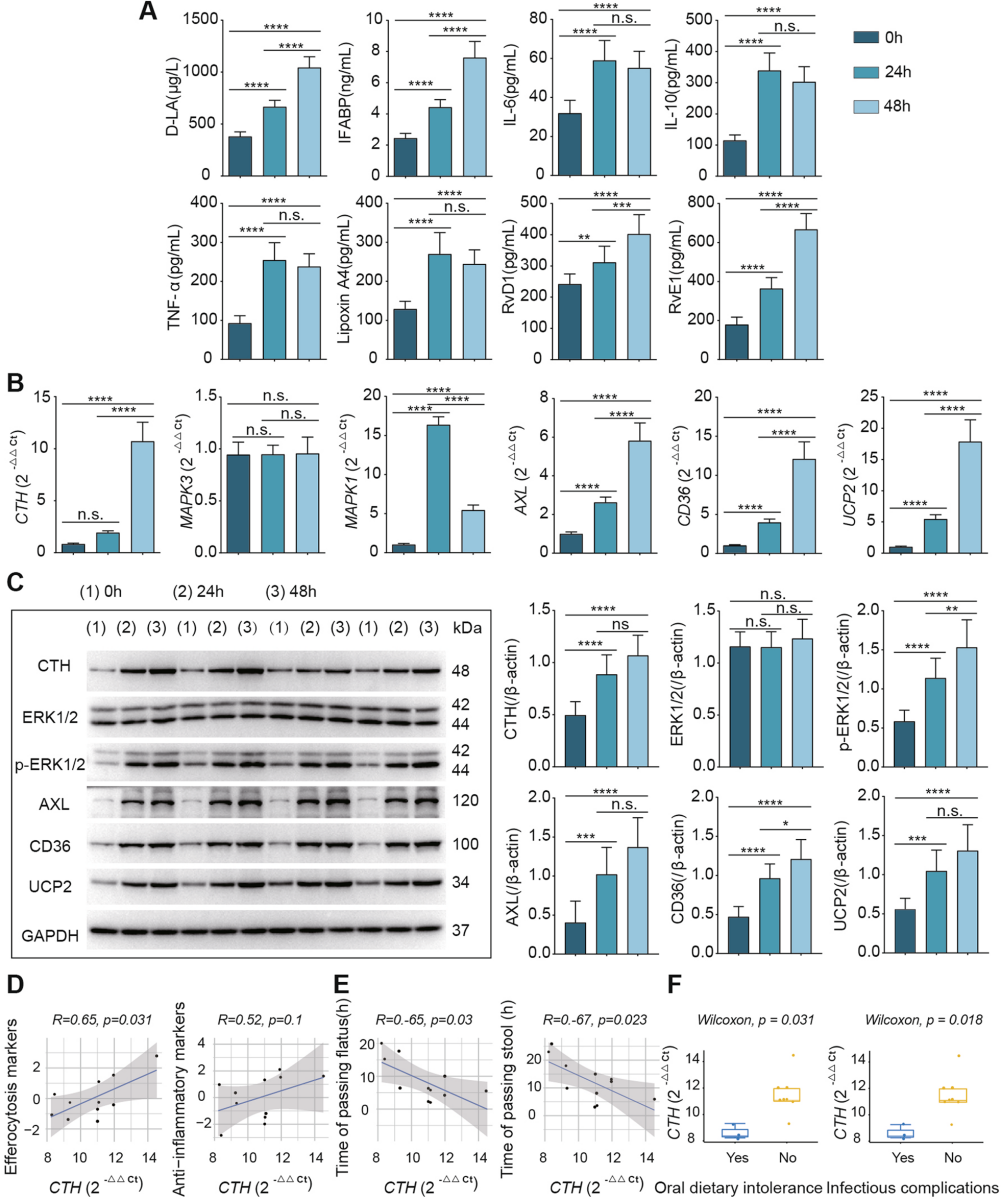
Clinical validation of changes in Cth, ERK1/2 and efferocytosis levels with the recovery of intestinal function and correlation analysis. **A** Postoperatively, the levels of intestinal barrier indicators (D-LA, IFABP), inflammatory factors (IL-6, TNF-α), anti-inflammatory factors (IL-10) and repair indicators (Lipoxin A4, RvD1, RvE1) changed as intestinal function was restored. **B, C** Postoperatively, changes in the gene levels of Cth, ERK1/2 and efferocytosis molecules (AXL, CD36, UCP2) were observed with prolonged intestinal recovery time. **D** Cth showed a positive correlation with efferocytosis biomarkers (AXL, CD36, UCP2) and anti-inflammatory repair indicators (IL-10, Lipoxin A4, RvD1, RvE1). **E** Correlation analysis of the mRNA levels of Cth at 48 h after surgery with the time of passing flatus and stool, suggesting that higher Cth levels were associated with less time for bowel function to resume. **F** Cth levels in patients with and without oral dietary intolerance at 48 h after surgery and in patients with and without infectious complications at 7 days after surgery. 0 h: the time of surgery; 24 h: 24 h after surgery; 48 h: 48 h after surgery. *P ≤ 0.05, **P ≤ 0.01, ***P ≤ 0.001, ****P ≤ 0.0001, one-way ANOVA with Tukey’s multiple comparisons test. The Wilcoxon rank sum test (two-group comparisons) or Pearson correlation (correlation tests) was used accordingly, and statistical significance was defined as *P* < 0.05

After demonstrating that Cth is dependent on the ERK1/2 pathway to regulate efferocytosis and induces an anti-inflammatory and repair phenotype in vivo and in vitro, clinical patients were sampled to assess the translational relevance of our results and to understand the severity of intestinal mucosal damage and repair after intestinal injury. Patient intestinal mucosal tissue was collected at the site of ileostomy intraoperatively, as well as 24 and 48 h postoperatively, via forceps biopsy, and patient blood was also collected. The anti-inflammatory factor IL-10 and repair-related SPMs (RvD1, RvE1, LipoxinA4) were expressed at progressively higher levels as the patients recovered from surgery (Fig. 8A). In addition, the gene and protein expression of Cth, AXL, CD36 and UCP2 in the intestinal mucosa, gradually increased with time, which was similar to the anti-inflammatory repair results. Moreover, the ERK1/2 pathway was gradually activated, and the phosphorylation level gradually increased (Fig. 8B, C). Specifically, the markers of intestinal barrier damage (D-LA and IFABP), as well as the proinflammatory molecules IL-6 and TNF-α, increased gradually over time, which may be the result of both damage and repair in the patient intestine.

### Cth correlates with efferocytosis and intestinal barrier repair in clinical patients

In addition to validating our findings in clinical patients, we also examined patient outcomes for the recovery of intestinal function, including the time of passing flatus and stool after surgery, oral dietary intolerance at 48 h after surgery, and infectious complications in patients 7 days after surgery [43]. Through exploratory observations, we found a significant positive correlation between Cth expression and efferocytosis markers at 48 h postoperatively (R = 0.65, *P* = 0.031), and Cth expression correlated positively with anti-inflammatory molecules. In addition, we also found a significant negative correlation between the level of Cth 48 h postoperatively and the length of time at which patients resumed defecation (R = − 0.65, *P* = 0.03) and bowel movements (R = − 0.67, *P* = 0.023), indicating that high expression of Cth was positively correlated with the recovery of intestinal function in patients. Finally, we found that the expression level of Cth was lower in patients with oral dietary intolerance at 48 h postoperatively and in patients with infectious complications at 7 days postoperatively. Overall, the patient data suggest that Cth and efferocytosis are closely related to the resolution of intestinal inflammation and barrier recovery in patients.

## Discussion

### Macrophage efferocytosis is involved in intestinal mucosal injury repair

To our knowledge, this is the first study showing that macrophage efferocytosis promotes intestinal mucosal injury repair in the context of I/R, further illustrating the role of macrophages in intestinal mucosal injury repair in I/R. Determining this specific role of macrophage efferocytosis in animal models is made difficult by the lack of effective markers that clearly distinguish between phagocytic and non-phagocytic states in macrophages. In several previous studies, different assays were designed according to the characteristics of animal models [4, 5]. In this study, we innovatively used labelled apoptotic endothelial cells pushed through an ostomy tube, observed macrophages engulfment of apoptotic endothelial cells by fluorescence microscopy and then calculated the efferocytosis index of macrophages, which enabled us to accurately understand the efferocytosis level of macrophages in a mouse model of I/R. In vitro, based on a previous study in which macrophages engulfed carboxylate beads as a control [4], we investigated whether macrophage engulfment of apoptotic endothelial cells was necessary to promote the emergence of a repair phenotype in macrophages.

It was found that the biomarkers of efferocytosis (AXL, CD36, UCP2) and macrophage repair indicators SPMs (RvD1, RvE1, LipoxinA4) were significantly increased in animal models and postoperative colorectal cancer patients. Moreover, in postoperative colorectal cancer patients, the biomarkers of efferocytosis and macrophage repair indicators significantly correlated with intestinal mucosal barrier function indicators, recovery indicators and inflammation indicators. In vitro, after simulating endothelial cell apoptosis due to ischaemia and hypoxia, macrophage engulfment of apoptotic endothelial cells was shown to be necessary for the emergence of a repair phenotype in macrophages, which was similar to previous findings of macrophage engulfment of other cells, such as Jurkat cells [16] and erythrocytes [4]. In addition, macrophages express many proinflammatory mediator-related genes (e.g., Hmgb1, CD86, Tnf, Cx3cr1) at the beginning of I/R and are involved in the pathological process of I/R. Over time, macrophages downregulate the expression of proinflammatory mediators and upregulate the expression of genes that inhibit the inflammatory response and haemoglobin degradation and promote efferocytosis and repair (e.g., IL-10, Hmox1, UCP2, and CD36).

Previous studies have shown that macrophages can play an important role in intestinal mucosal injury repair through the production of factors that promote proliferation and repair. For example, macrophage-derived IL-10 promotes intestinal mucosal endothelial cell proliferation and intestinal mucosal injury repair by regulating the WISP-1 signalling pathway [22]. MiR-590-3p, a factor in M2 macrophage-derived exosomes, reduces inflammatory signalling and promotes epithelial regeneration by targeting LATS1 in colitis mice [44]. The STAT6-dependent macrophage phenotype promotes mucosal repair in TNBS-treated (2,4,6-trinitrobenzenesulfonic acid, TNBS) mice through activation of the Wnt signalling pathway [45]. However, the relationship between efferocytosis by macrophages and intestinal barrier repair has rarely been reported, and only one recent study focused on the repair of the intestinal mucosal barrier by macrophage efferocytosis. Using a murine model of intestinal barrier damage due to chronic inflammation induced by a cholic acid-containing high-fat diet, investigators found that macrophage COX2 could mediate efferocytosis, resolution reprogramming, and intestinal epithelial repair [10]. Thus, our study provides a novel theoretical basis for the involvement of macrophage efferocytosis in intestinal mucosal injury repair.

### Cth is involved in macrophage efferocytosis and intestinal mucosal repair

In this study, we identified and validated the involvement of Cth in macrophage efferocytosis and the promotion of intestinal mucosal repair in the context of I/R by performing transcriptome sequencing analysis of macrophages at different time points after I/R. Previous studies have focused on the role of Cth in the repair of intestinal barrier damage due to intestinal inflammation, and a small number of clinical studies showed that reduced expression of cystathionine-β-synthase (CBS) and Cth in patients with Hirschsprung disease may affect mucosal integrity and colonic contractility, which are possible causes of hirschsprung-associated enteritis [14]. Another study in a murine model of colitis showed that Cth could treat colitis by affecting H_2_S synthesis, correcting microbiota biofilm dysregulation and mucus layer reconstruction [13]. In addition, in a mouse model of gastrointestinal mucosal injury induced by acetyl salicylic acid and NSAIDs, Cth was shown to mediate FXR production to protect the gastrointestinal mucosal barrier [15]. However, no studies have focused on whether Cth is involved in intestinal mucosal repair by mediating macrophage efferocytosis.

### Cth promotes macrophage efferocytosis and intestinal mucosal repair by activating ERK1/2 phosphorylation

In this study, clinical validation section showed that the expression of Cth and p-ERK increased significantly with the recovery of intestinal function, and both factors showed a positive correlation. In vivo, Cth could promote macrophage efferocytosis, attenuate the inflammatory response and promote intestinal mucosal repair, all of which were dependent on the ERK1/2 signalling pathway. Similarly, in vitro, Cth was dependent on the ERK1/2 signalling pathway to promote macrophage efferocytosis (CD36, Axl, UCP2) and macrophage repair phenotypes (RvD1, RvE1, Lipoxin A4) and reduce inflammatory responses (CD86, IL-10).

It has been previously demonstrated that Cth regulates the ERK1/2 signalling pathway through the production of H_2_S, which in turn regulates cell proliferation [12, 19] and inflammatory responses [46], and this is the main reason why H_2_S was not detected or targeted in this study.

The ERK1/2 signalling pathway has been reported to play an immunomodulatory role in tumorigenesis [47], reproduction [48] and oxidative stress [49]. It has been shown that the clearance of apoptotic cells by macrophages is involved in the production of the pro-resolving mediators PGE2 and TGF-β1 through epigenetic repression of the ERK1/2 phosphatase Dusp4 [17]. In addition, macrophage migration contributes to the clearance of apoptotic cells, which is a crucial step in resolving inflammation and restoring homeostasis in vivo. Recent studies have also shown that ERK1/2 is involved in the migration of macrophages during efferocytosis [18]. Similar results were obtained in our study; ERK1/2 promoted efferocytosis (Axl, CD36, UCP2), which in turn inhibited inflammation (IL-6, TNF-α, IL-10) and promoted intestinal mucosal barrier (D-LA, IFABP) repair in the I/R model.

## Conclusions

**Fig. 9:**
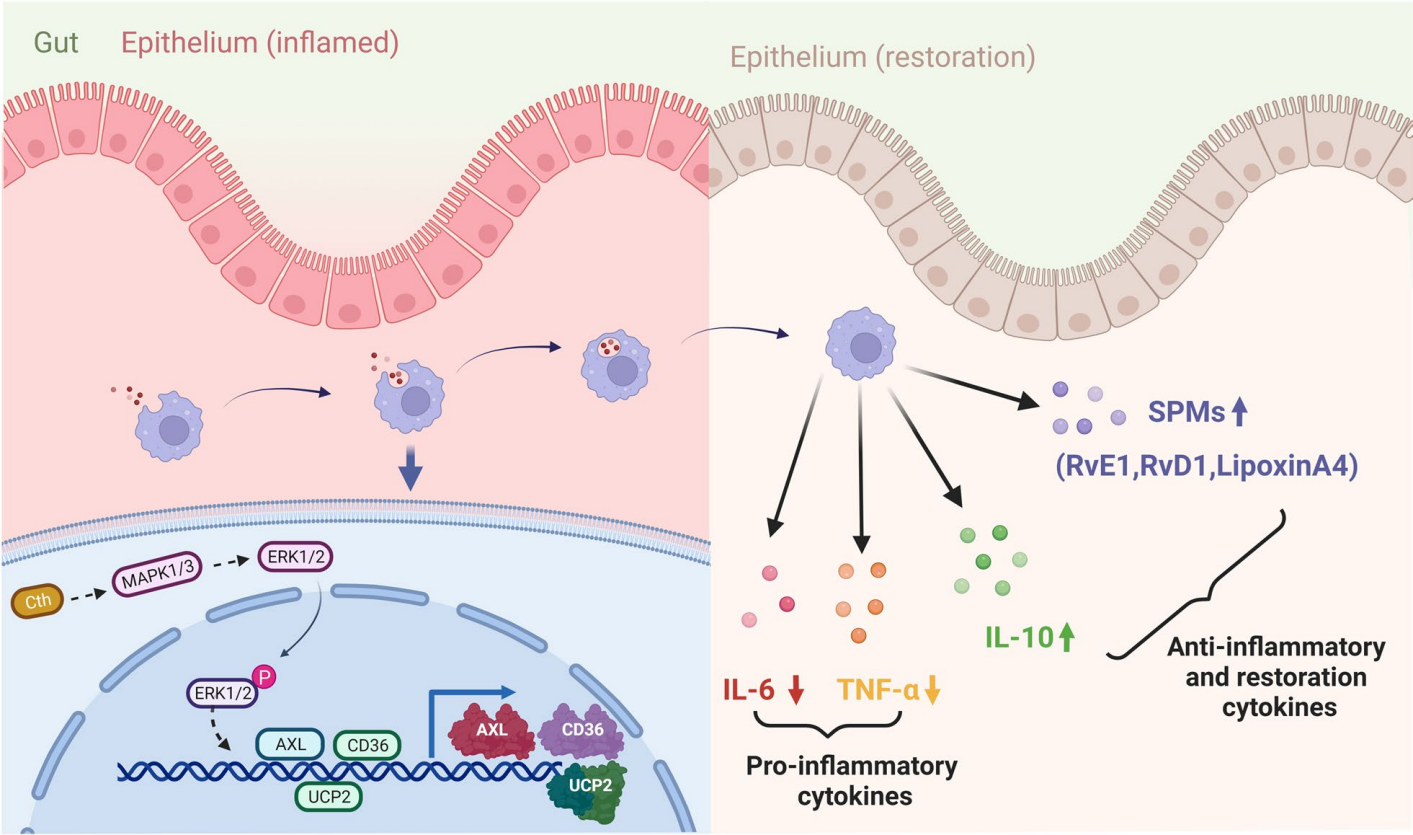
Schematic demonstrating that Cth can activate the ERK1/2 signalling pathway, induce macrophage efferocytosis, and promote intestinal barrier repair. Macrophages are recruited to the site of intestinal injury, and Cth activates the ERK1/2 signalling pathway in macrophages, which in turn induces macrophage phagocytosis of apoptotic endothelial cells, inhibits the release of the proinflammatory factors IL-6 and TNF-α, and promotes the secretion of SPMs (Lipoxin A4, RvD1, RvE1) and the anti-inflammatory factor IL-10, thereby helping to restore intestinal barrier function

Although research on intestinal mucosal injury repair has been conducted for decades, enteral nutrition and glutamine can improve intestinal mucosal barrier function and promote intestinal mucosal injury repair to some extent, and immunomodulatory drugs are also a key direction of interest for researchers. However, an in-depth study of the molecular mechanism of immunomodulation is needed before these treatments can be applied in the clinic. In this study, we found that the Cth-dependent ERK1/2 pathway induced macrophage efferocytosis, which in turn regulated intestinal mucosal injury repair (Fig. 9). Therefore, manipulating efferocytosis to promote intestinal mucosal injury repair may be an effective strategy. Moreover, the findings of this study provide new clues to further clarify the immune mechanisms affecting intestinal mucosal injury repair, which are important for promoting the recovery of patients with I/R.

## Materials and methods

### Mice

Male C57BL/6 mice (6–8 weeks, 18–22 g) were purchased from Kunming Medical University [SCXK(Dian)k2015-0002] and Hunan Sja Laboratory Animal Co., Ltd. [SCXK(Xiang)2019-0004], and all mice were housed in stainless steel cages with controlled indoor temperature and humidity. The mice were provided ad libitum access to water and standard mouse food during the week prior to the surgery. All animal experiments were performed according to the experimental animal care and use methods published by the Ministry of Science and Technology of China and approved by the Ethics Committee of Kunming Medical University.

### Animal model

I/R model: Before the experiment, all instruments were sterilized and put into the oven to dry, medical alcohol (75%) was sprayed on the workbench, and the experiment was started after the alcohol evaporated and workbench was dry. The mice were anaesthetized with 3% chloral hydrate at a dose of 0.15 ml/10 g. The abdomen was shaved, the skin was disinfected with iodophor, a median abdominal incision was made, the caecum and the ileum at the upper part of the caecum were removed from the abdominal cavity with a cotton swab and placed on a saline-wetted gauze block. The superior mesenteric artery (SMA) root vessels were bluntly separated, the vessels were tied closed for 30 min, and the knot was untied to restore blood flow to induce ischaemia‒reperfusion. Sham model: The SMA was bluntly separated but not ligated, and the rest of the steps were similar to those above.

### In vivo efferocytosis assay

After the animal model was established, an ostomy tube was left in the jejunal segment, anastomosed and fixed to the intestinal and abdominal walls, the intestine was returned to the abdominal cavity, and the muscle and skin were sutured layer by layer (Fig. 2A). Then, labelled apoptotic endothelial cells were injected through the ostomy tube into the intestine, and the mice were sacrificed after 3 h. The small intestinal mucosa and fluid were collected, and the engulfment of cells was subsequently observed by fluorescence microscopy. At least three different fields per sample were analysed, and at least 30 macrophages per field were evaluated [5].

### Mouse groupings and interventions

Part 1: Thirty C57BL/6 male rats were randomly divided into 5 groups of 6 rats each, all of which were divided into the 12 h post-I/R, 24 h post-I/R, 36 h post-I/R, 48 h post-I/R and sham surgery groups. At the corresponding time points, three mice in each group were sacrificed, and intestinal mucosa tissue was taken for analysis. The other three mice were injected with labelled apoptotic endothelial cells via the ostomy tube into the intestine, and the mucosa and small intestinal fluid were collected after 3 h to observe efferocytosis.

Part 2: Thirty-six C57BL/6 male rats were randomly divided into 6 groups of 6 rats each, and the mice were divided into the sham-operated group; I/R group; I/R + Cth inhibitor group; I/R + Cth agonist group; I/R + Cth agonist + ERK inhibitor group; and I/R + Cth inhibitor + ERK agonist group. The sham-operated and I/R groups were fed normally at the end of surgery; the I/R + Cth inhibitor group was given 50 mg/kg propargylglycine (PAG) [38] intraperitoneally at the end of the surgery. The I/R + Cth agonist group was given 50 mg/kg methacholine (MCH)[37] intraperitoneally at the end of the surgery. In the I/R + Cth agonist + ERK1/2 inhibitor group, 50 mg/kg MCH was injected intraperitoneally at the end of the surgery, followed by 10 mg/kg PD98059 (PD) [40, 41] after full absorption; in the I/R + Cth inhibitor + ERK1/2 agonist group, 50 mg/kg PAG was injected intraperitoneally at the end of the surgery, followed by 100 µg/kg PMA [39] after full absorption. The efficiency of the inhibitors and agonists were showed in Additional file 6: Figure S6. Three mice in each group were sacrificed, and intestinal mucosa tissue was taken for analysis. The other three mice were injected with labelled apoptotic endothelial cells via the ostomy tube into the intestine, and the mucosa and small intestinal fluid were collected after 3 h to observe efferocytosis.

### Isolation and collection of intestinal mucosal macrophages

The intestinal mucosa was obtained from each group of mice after sacrifice; the intestinal mucosa tissues were washed in D-Hank’s buffer and then placed in medium with 10 ml of 10 mM DTT solution for 30 min to remove mucus at 4 °C. Then, the cells were placed in 10 ml of 0.5 mM EDTA solution in a 37 °C water bath shaker for 30 min to remove the epithelium, and this step was repeated 2 times until the solution was free of epithelial cells (after EDTA treatment, the tissue became white and had a nearly transparent lamellar structure). Then, the samples were transferred to a Petri dish containing 10 ml of enzyme solution (collagenase 1 mg/ml, DNase I 0.01%, HEPES 25 mM). Then, the samples were placed into a dish and incubated in 5% CO_2_ at 37 °C for 3–4 h and mixed several times every hour with a syringe. The filtrate was collected by centrifugation at 1500 RPM for 8 min, and the supernatant was removed. The cells were with 4 ml of 40% Percoll, and 4 ml of 70% Percoll was slowly added above the liquid surface and centrifuges at 1500 RPM for 25 min (centrifugal deceleration set to 0) at 18–22 °C. Approximately 1–2 ml of cells was carefully aspirated, phosphate buffer (PBS) was added to wash the precipitate, the cells were centrifuged at 1500 RPM for 8 min, and the supernatant was thoroughly removed. The cells were resuspended, and cell counting was performed using flow cytometry to screen macrophages before using CD11b and F4/80 antibodies to label the cells. The sorted phagocytes were divided into two groups, one for transcriptome analysis and one for RNA extraction for PCR.

### Transcriptome analysis

Total RNA was collected from macrophages in the mouse intestinal mucosa after sorting and extraction using TRIzol reagent (Invitrogen, CA, USA) according to the manufacturer's procedure. Total RNA quantity and purity were analysed with a Bioanalyzer 2100 and RNA 6000 Nano LabChip Kit (Agilent, CA, USA) with a RIN > 7.0. Approximately 10 µg of total RNA was used to isolate poly(A) mRNA with poly-T oligo attached magnetic beads (Invitrogen). Indexed libraries were pooled and sequenced using an Illumina Novaseq 6000. Paired-end 150 bp reads were generated per sample. The results of transcriptome sequencing analysis are shown in Additional file 8: Table S1.

### Culture and acquisition macrophages

The macrophages that were cocultured with apoptotic endothelial cells were divided into two groups. Class 1 cells were mouse bone marrow-derived macrophages. The mice were selected at 6–8 weeks of age, executed, and sterilized by soaking in 75% ethanol, and their femoral tibial bone marrow cells were collected in a centrifuge tube at 1300 r/min for 3 min. After being lysed with erythrocyte lysis solution, the bone marrow cells were resuspended in culture medium, evenly inoculated in 10 cm culture dishes and incubated overnight to remove other miscellaneous cells, such as fibroblasts, which adhere to the wall more quickly. The nonadherent cells were collected and inoculated in new culture plates, and 50 ng/ml recombinant murine M-CSF was added and incubated for 7 days to produce bone marrow-derived macrophages (the medium contained α-MEM medium + 10% FBS + 1% double antibodies). The viability and morphology of the cells were observed by inverted microscopy on days 1, 3 and 6, including the state of apposition, changes in volume, morphology and pseudopods (photographed and recorded). Flow double-label identification was performed using F4/80 and CD11b antibodies for subsequent experiments. Class 2 cells were THP-1 cells that were stimulated with 10 ng/ml PMA for 72 h to induce them into macrophages, followed by flow double-label identification using CD11b and CD14 for subsequent experiments.

### Culture and acquisition of apoptotic endothelial cells

Hypoxia–ischaemia was used to induce apoptosis in endothelial cells [50]. To simulate hypoxia, the specimens were placed in a specially designed sealed chamber system (Mitsubishi Gas Chemical Co., INC, Japan). To simulate ischaemia, the normal culture medium was replaced with the same volume of sugar-free calcium-containing PBS. To simulate ischaemia-hypoxia, the previously described procedures were performed simultaneously. The intervention time was 2 h. Caco-2 (Procell) and IEC-6 cells (Feng Hui Sheng Wu) were placed in ischaemic and hypoxic environments for 2 h, respectively, and the cells were collected and stained using Annexin V to stain and flow cytometry to calculate the apoptosis rate [5] to determine the optimal apoptotic environment for endothelial cells.

### Flow cytometric detection of apoptosis

Caco-2 and IEC-6 cells were detached with 0.25% trypsin (Gibco 1894145), and 400 µl binding buffer was used to resuspend the cells. The cells were dived into 4 tubes: the first tube was a control tube (without any reagent), the second tube was a FITC control tube (5 µl Annexin V FITC solution), the third tube was a PI control tube (10 µl PI solution), and the fourth tube was used as the experimental tube (5 µl Annexin V FITC solution and 10 µl PI solution). The cells were incubated for 15 min at room temperature in the dark. Then, 1 ml of PBS was added, and apoptosis was detected by flow cytometry. The kits were purchased from Dojindo Laboratories (AD10), and the results were analysed and presented using FlowJo software.

### In vitro efferocytosis assay

Apoptotic endothelial cells were added to macrophages (human-derived macrophages cultured with human-derived endothelial cells and murine-derived macrophages cultured with murine-derived endothelial cells), and hydroxylate beads were used as controls. Red fluorescent CellTracker™ Deep Red Dye (Invitrogen C34565) was used to label macrophages, and green fluorescent CellTracker™ Green BODIPY™ Dye (Invitrogen C2102) was used to label endothelial cells and hydroxylate beads (Sigma L5155-1ML). Four groups were included: the human macrophages + human apoptotic Caco-2 cell group, human macrophages + carboxylate bead group, mouse macrophages + mouse apoptotic IEC-6 cell group and mouse macrophages + carboxylate bead group. After eluting the unengulfed endothelial cells, engulfed cells were observed with a fluorescence microscope (Olympus CKX41).

### ELISA

ELISA kits for RvD1, RvE1, Lipoxin A4, I-FABP, D-LA, IL-6, IL-10 and TNF-α were purchased from Mlbio. The damaged intestinal mucosa, serum, and cells were processed, and the lysates were used for ELISA according to the manufacturer's instructions.

### q-PCR

The samples were lysed using TRIzol reagent (Lifetech 15596026), and the concentration of RNA in DEPC in the kit was measured with a UV spectrophotometer (DeNovix or NanoDropND-1000). The volume of total RNA required for reverse transcription was calculated, and the first-strand cDNA was synthesized according to the instructions of the FastKing RT Kit (With gDNase). After target gene was amplified by a quantitative PCR instrument (TL988 or LightCycle 96), Ct value analysis was performed. In this study, the q-PCR primers were designed using Beacon Designer 7.90, and the primer sequences are shown in Additional file 9: Table S2 (F: stands for upstream primer, FORWARD; R: downstream primer, REVERSE).

### Western blot analysis

Tissue was placed in 10:1 RIPA lysis buffer (Beyotime) and protease inhibitor (Roche) to obtain the supernatant, and the protein concentration was determined with a BCA protein concentration assay kit (Beyotime). Equal amounts of protein (80 μg or 30 μg protein per sample lane) were subjected to 4–10% SDS‒PAGE (BIO-RAD) and transferred to 0.45 μm PVDF membranes (Millipore). The membranes were then blocked with 5% BSA (Beijing Solarbio Science & Technology Co., Ltd.), rinsed 3 times with TBST and placed in a refrigerator at 4 °C overnight with the primary antibody. The primary antibody was removed, the membrane was rinsed 3 times, and the secondary antibody was added and incubated for 2 h at room temperature (see Additional file 9: Table S2 for antibodies). After the membrane was rinsed again, ECL chromogenic solution (Millipore) was added for colour development, and images were acquired using a gel imager (Tanon). The relative densities of the Cth, ERK1/2, p-ERK1/2, Axl, CD36 and UCP2 protein bands were normalized to β-actin. Expression was quantified as density units using Image Lab 6.0 software.

### Haematoxylin and eosin (H&E) staining

The intestinal mucosa from mice was embedded in paraffin. Transverse sections (5 μm) were obtained and stained with H&E. The slices were evaluated by microscopy (Danjier, China) with 3DHISTECH Slide Converter, and CaseViewer 2.4 was used to evaluate and save images (scale = 50 μm).

### Immunofluorescence analysis

Paraffin-embedded intestinal mucosa sections (5 μm) were immersed in citrate buffer (pH 6.0), boiled for 10 min, cooled naturally, and then blocked with 5% sheep serum for 60 min at room temperature. After shaking off the excess blocking solution, anti-cth (1:450) and anti-F4/80 (1:200) primary antibodies were added and incubated overnight at 4 °C. After 4 rinses with PBST, Affinuty purified Antibody Daylight 488-labelled goat anti-Rabbit IgG (H + L) (corresponding to anti-cth, dilution ratio 1:1000) and Daylight 549-conjugated Affinipure goat Anti-Mouse IgG, Light chain specific (corresponding to primary antibody anti-F4/80, dilution ratio 1:800) secondary antibodies were added. After 3 rinses with PBST, p-ERK1/2–AF647 (1:100) antibody was added and incubated at 37 °C for 1 h. After 3 washes to remove excess solution, 50 µl of blocker (containing DAPI) was added to seal the slices. The slices were photographed under a laser confocal fluorescence microscope (Olympus FV1000). Additional file 9: Table S2 contains information on the antibodies used.

### Clinical samples and ethics statement.

A pilot observational study was designed to verify the findings. Patients with colorectal cancer requiring prophylactic ileostomy were recruited prospectively from December 2020 to November 2022 from the Department of Gastrointestinal Surgery at The Second Affiliated Hospital of Kunming Medical University. The inclusion criteria were as follows: 1. patients aged 40–85 years; 2. patients with colorectal cancer and partial obstruction or patients with ultralow rectal cancer; and 3. patients undergoing laparoscopic radical resection for colorectal cancer and requiring prophylactic ileostomy according to intestinal conditions during operation. The exclusion criteria were as follows: 1. pregnancy; 2. total colorectal obstruction; 3. prior history of major abdominal surgery; and 4. severe abdominal infection. In addition, all patients provided informed consent, and the study was conducted in accordance with the guidelines and was approved by the Ethics Committee of Second Affiliated Hospital Kunming Medical University (PJ-2020-138, PJ-2021-212).

### Clinical samples and patient information collection.

Due to the condition of the bowel and to prevent the occurrence of anastomotic leakage, a loop of ileum was pulled out through the incision in the abdomen after laparoscopic radical resection for colorectal cancer. A 3–4 cm cut was excised from the ileum to form the site stoma. The incision was then intermittently sutured during the surgery and reopened at 24 and 48 h postoperatively for intestinal fluid drainage and stoma bag attachment, respectively. Patient intestinal mucosal tissue was collected at the site of ileostomy intraoperatively, as well as 24 and 48 h postoperatively, via forceps biopsy. Each mucosal tissue specimen was split into two samples. One of the samples, which was used for q-PCR and western blotting, was snap frozen immediately using liquid nitrogen and stored at − 80 °C. The other samples were used for immunohistochemical staining and were fixed using 4% paraformaldehyde and embedded in paraffin. Patient venous blood was sampled intraoperatively and 48 h postoperatively. Serum was isolated and stored at − 80 °C for subsequent molecular testing. Finally, the patients were followed up for postoperative recovery outcomes, including oral dietary tolerance at 48 h after surgery, the time of passing flatus and stool, and the occurrence of infectious and noninfectious complications on the 7th day postsurgery (Additional file 10: Table S3).

### Statistical analysis

All bar graphs are expressed as the mean ± standard deviation. Data between two groups were compared using Student's t test. Comparisons between multiple groups were made using one-way ANOVA with Tukey’s test. Graphs were plotted using GraphPad Prism 8.0, and *P* values less than 0.05 were considered significant. The Wilcoxon rank sum test (two-group comparisons) or Pearson correlation (correlation tests) was used accordingly. Statistical significance was defined as *P* < 0.05.

## Supplementary Information

**Additional file 1**: **Fig. S1**. Preparation of apoptotic endothelial cells and macrophages.** A** Determining the conditions foroptimal apoptosis rates in intestinal endothelial cells using flow cytometry.** B** Primary culture and identification ofmouse bone marrow-derived macrophages. Double-labelling with F4/80 and CD11bantibodies and flow cytometric identification of macrophage purity.** C** THP-1 cells were induced to differentiate into macrophages. Flowdouble-label identification using CD11b and CD14 antibodies was performed.

**Additional file 2**: **Fig. S2**. Flow cytometric analysis of changes in the intestinal efferocytosis index in a live animal model.

**Additional file 3**: **Fig. S3**. Construction of a stable strain of macrophage Cth overexpression/silencing vectors.*P ≤ 0.05, **P ≤ 0.01, ***P ≤ 0.001, ****P ≤ 0.0001, one-way ANOVA with Tukey’smultiple comparisons test.

**Additional file 4**: **Fig. S4**. Excluding the effect of Cth silencing or overexpression onmacrophage viability using flow cytometry.

**Additional file 5**: **Fig. S5**. The effect ofthe ERK1/2 inhibitor PD98059, the ERK1/2 agonist PMA, and the efferocytosisinhibitor cytochalasin D on cell viability was excluded using flow cytometry.

**Additional file 6**: **Fig. S6**. Exploration ofoptimal effector doses of Cth inhibitor propargylglycine (PAG), Cth agonistmethacholine (MCH), ERK1/2 inhibitor PD98059 (PD) and ERK1/2 agonist PMA in themodel of I/R. n=3/group.

**Additional file 7**: **Fig. S7**. Representativefluorescence images and the relatedindex of efferocytosis in vivo functional validation experiments.

**Additional file 8**:** Table S1**. The results of transcriptome sequencing analysis.

**Additional file 9**:** Table S2**. qPCR primers and antibodies used in this study.

**Additional file 10**:** Table S3**. Patient’s clinical information.

## Data Availability

The data generated during and/or analyzed during the current study are available from the corresponding author on reasonable request. The results of transcriptome sequencing analysis are shown in Additional file 8: Table S1.

## Abbreviations

Cth: Cystathionine gamma-lyase
I/R: Intestinal ischaemia‒reperfusion injury
ERK1/2: Extracellular signal-regulated kinase 1/2
COX2: Cyclooxygenase 2
Hmox1: Haem oxygenase-1
Clec7a: Dectin-1
SPMs: Specialized pro-resolving mediators
PCA: Principal component analysis
DEGs: Differentially expressed genes
MAPK: Mitogen-activated protein kinase
I-FABP: Intestinal fatty acid binding protein
CBS: Cystathionine-β-synthase
SMA: Superior mesenteric artery
PAG: Propargylglycine
MCH: Methacholine
PD: PD98059
