# Supplementary figures and images for: Cystathionine gamma-lyase (Cth) induces efferocytosis in macrophages via ERK1/2 to modulate intestinal barrier repair
Source: Cell Commun Signal. 2023 Jan 23;21:17. doi: 10.1186/s12964-022-01030-y (PMC9869634; doi:10.1186/s12964-022-01030-y)

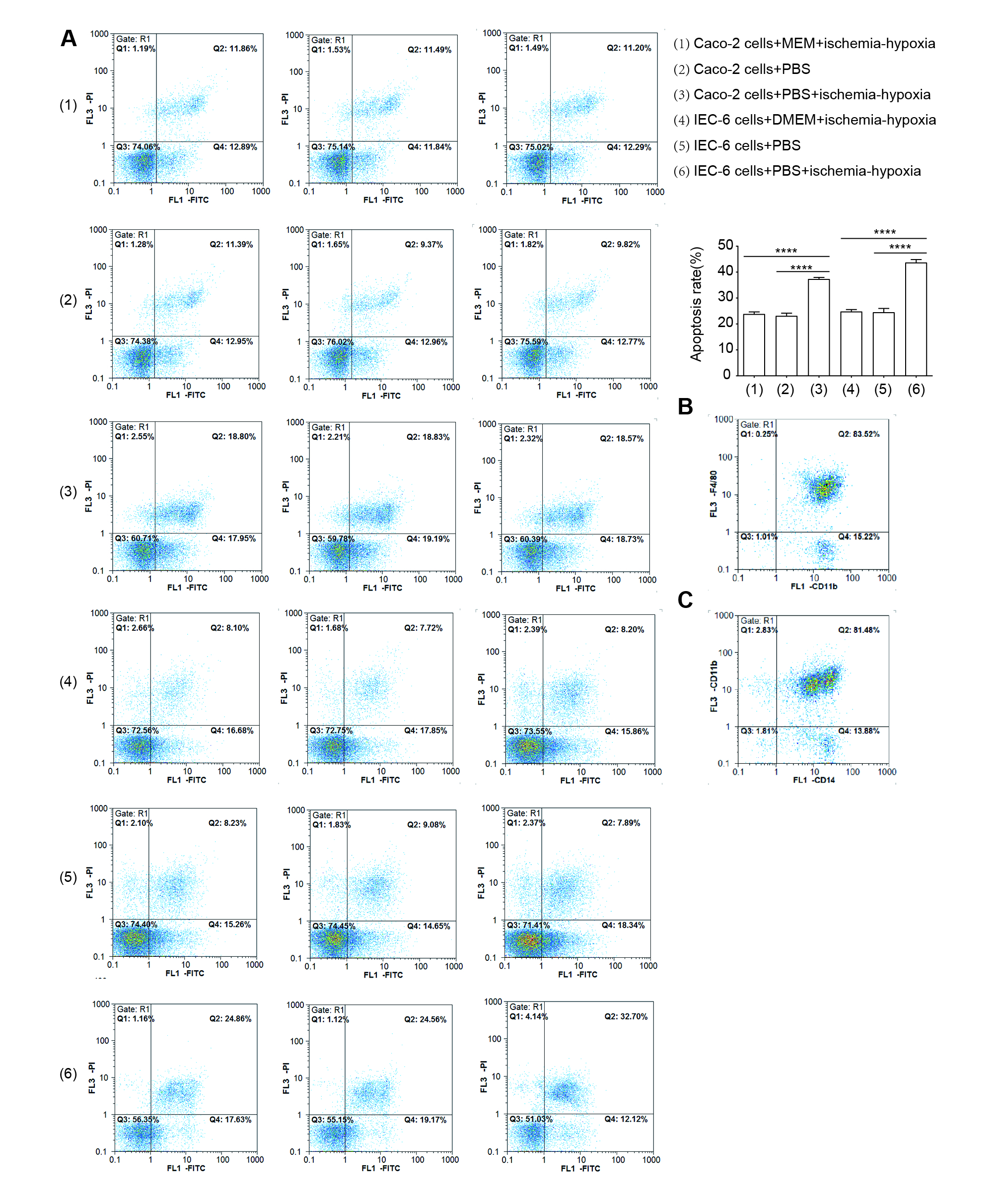

Supplement: Supplementary file 2 — Additional file 1: Fig. S1. Preparation of apoptotic endothelial cells and macrophages. A Determining the conditions foroptimal apoptosis rates in intestinal endothelial cells using flow cytometry. B Primary culture and identification ofmouse bone marrow-derived macrophages. Double-labelling with F4/80 and CD11bantibodies and flow cytometric identification of macrophage purity. C THP-1 cells were induced to differentiate into macrophages. Flowdouble-label identification using CD11b and CD14 antibodies was performed. [file 12964_2022_1030_MOESM2_ESM.jpg]

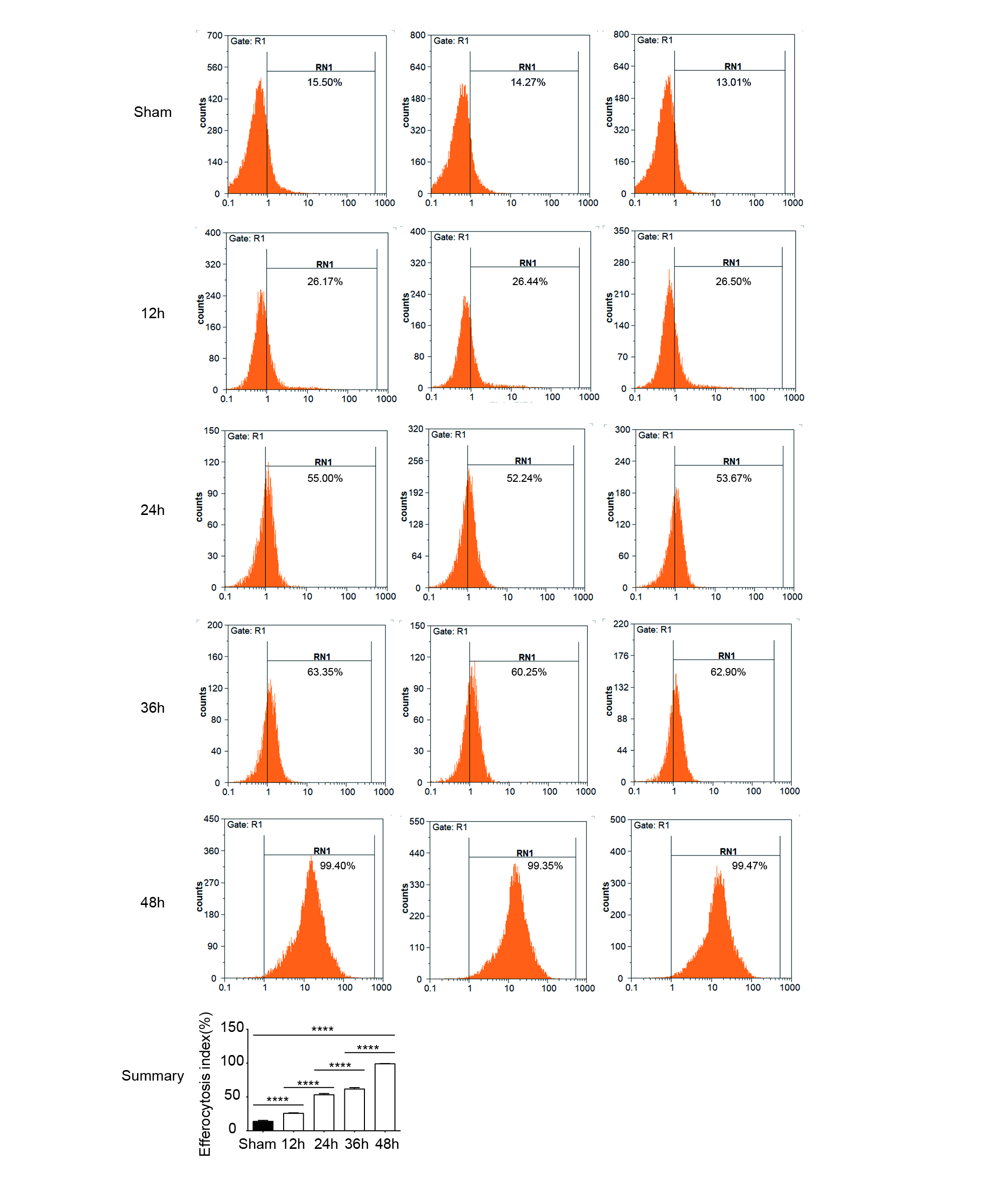

Supplement: Supplementary file 3 — Additional file 2: Fig. S2. Flow cytometric analysis of changes in the intestinal efferocytosis index in a live animal model. [file 12964_2022_1030_MOESM3_ESM.jpg]

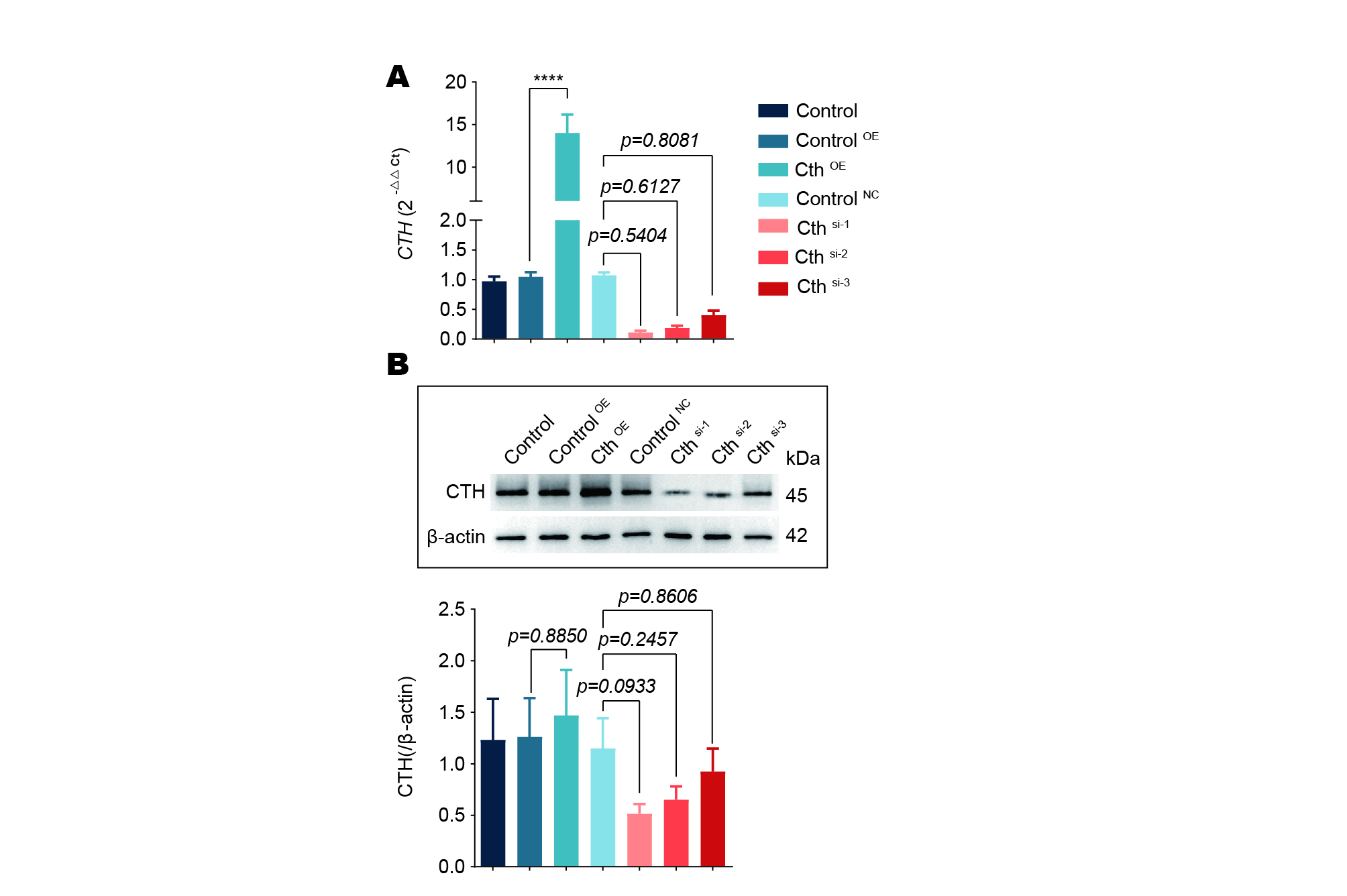

Supplement: Supplementary file 4 — Additional file 3: Fig. S3. Construction of a stable strain of macrophage Cth overexpression/silencing vectors.*P ≤ 0.05, **P ≤ 0.01, ***P ≤ 0.001, ****P ≤ 0.0001, one-way ANOVA with Tukey’smultiple comparisons test. [file 12964_2022_1030_MOESM4_ESM.jpg]

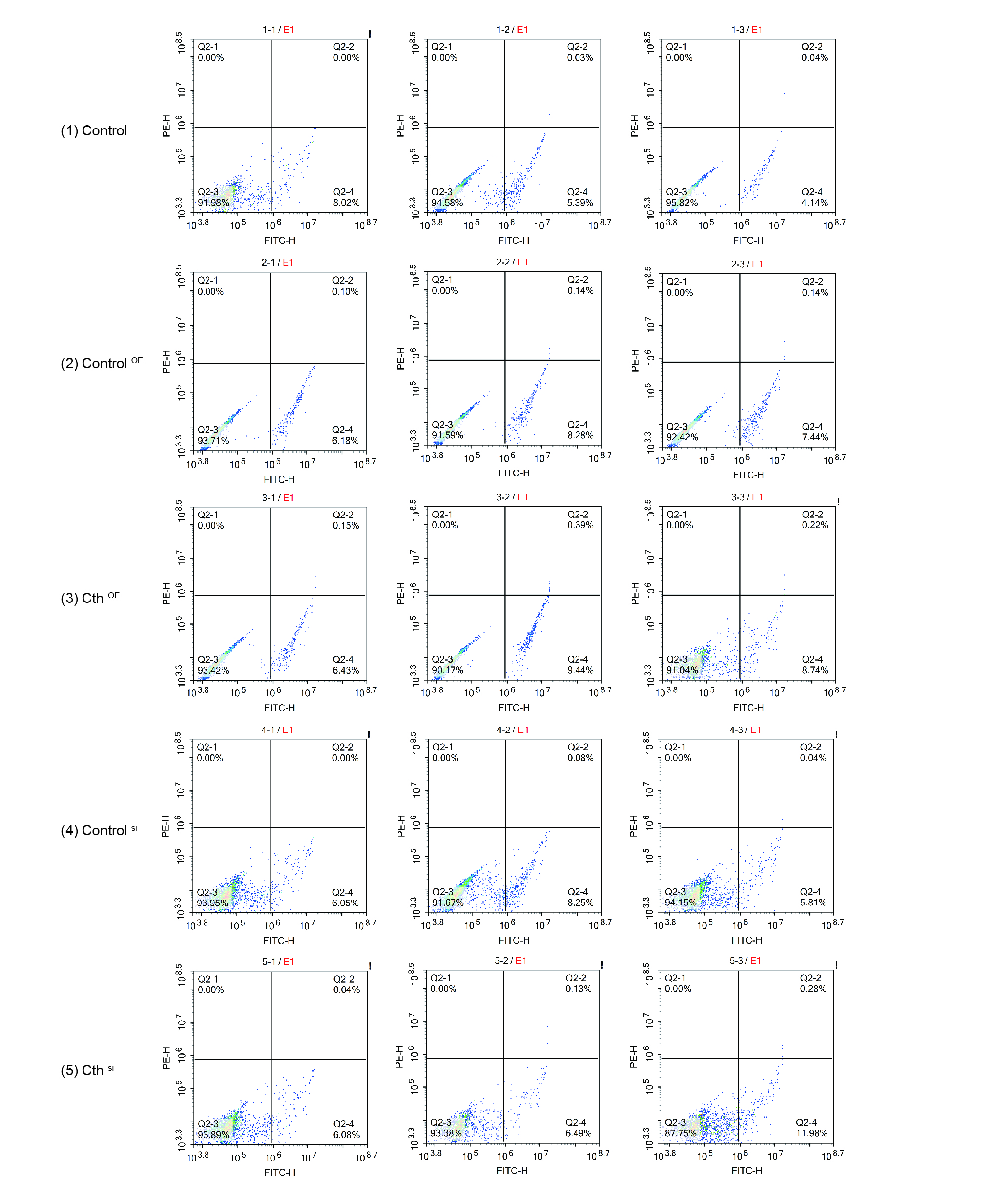

Supplement: Supplementary file 5 — Additional file 4: Fig. S4. Excluding the effect of Cth silencing or overexpression onmacrophage viability using flow cytometry. [file 12964_2022_1030_MOESM5_ESM.jpg]

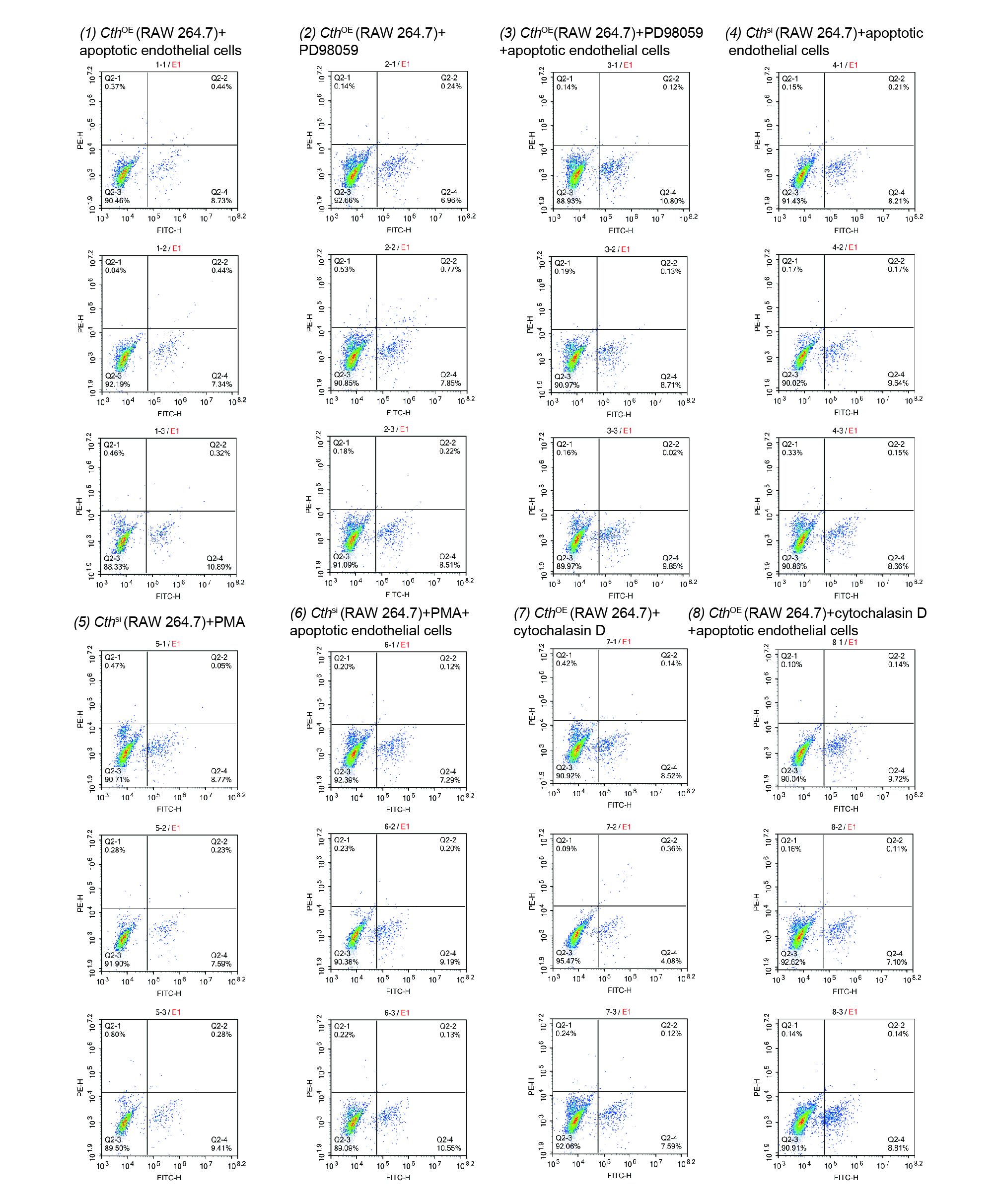

Supplement: Supplementary file 6 — Additional file 5: Fig. S5. The effect ofthe ERK1/2 inhibitor PD98059, the ERK1/2 agonist PMA, and the efferocytosisinhibitor cytochalasin D on cell viability was excluded using flow cytometry. [file 12964_2022_1030_MOESM6_ESM.jpg]

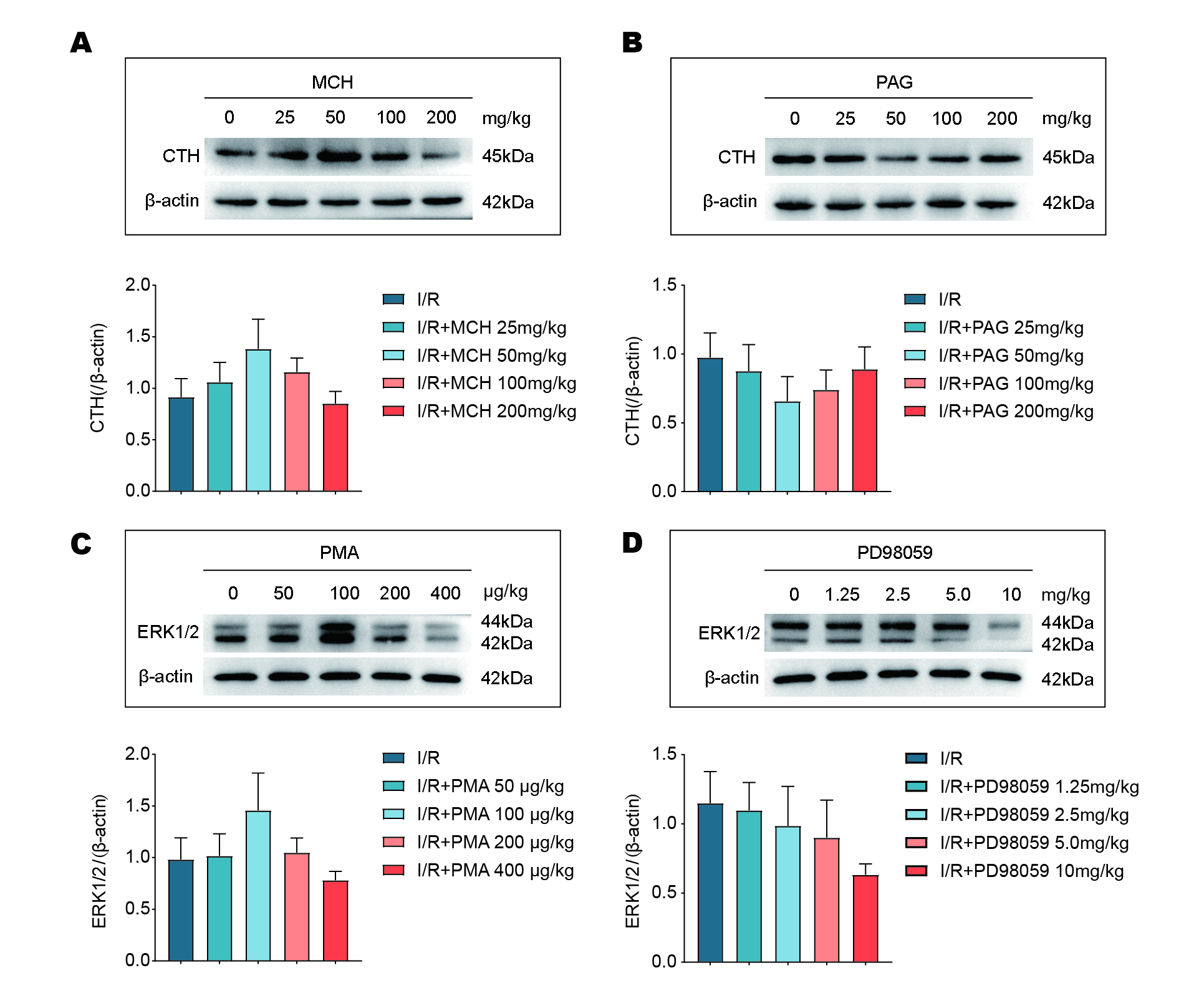

Supplement: Supplementary file 7 — Additional file 6: Fig. S6. Exploration ofoptimal effector doses of Cth inhibitor propargylglycine (PAG), Cth agonistmethacholine (MCH), ERK1/2 inhibitor PD98059 (PD) and ERK1/2 agonist PMA in themodel of I/R. n=3/group. [file 12964_2022_1030_MOESM7_ESM.jpg]

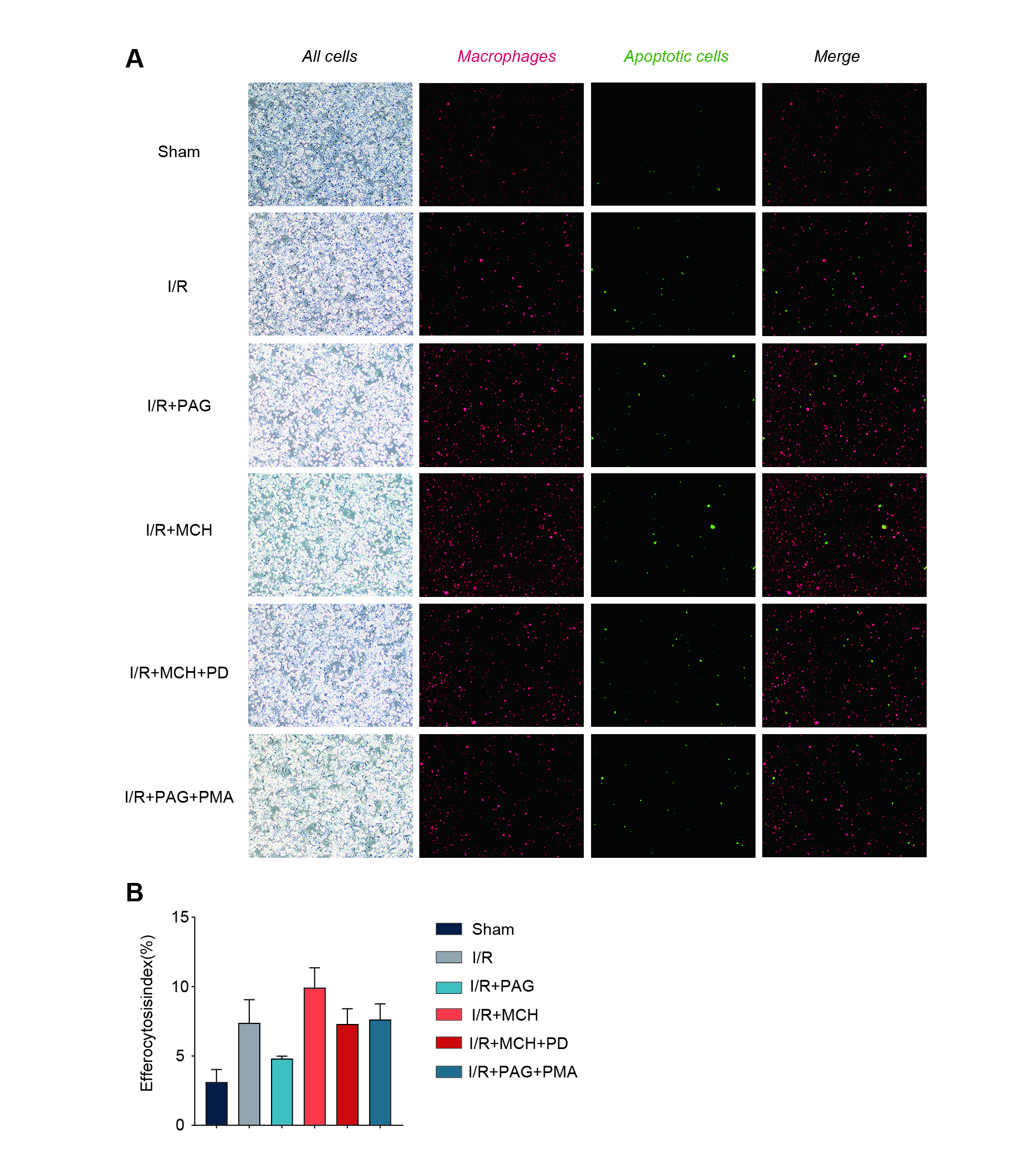

Supplement: Supplementary file 8 — Additional file 7: Fig. S7. Representativefluorescence images and the relatedindex of efferocytosis in vivo functional validation experiments. [file 12964_2022_1030_MOESM8_ESM.jpg]
